# Schwann cell-derived extracellular vesicles promote memory impairment associated with chronic neuropathic pain

**DOI:** 10.1186/s12974-024-03081-z

**Published:** 2024-04-17

**Authors:** Yidan Tang, Jiahui Wu, Changliang Liu, Lu Gan, Hai Chen, Ya-Lan Sun, Jin Liu, Yuan-Xiang Tao, Tao Zhu, Chan Chen

**Affiliations:** 1 Department of Anesthesiology, West China Hospital, Sichuan University, Chengdu, Sichuan 610041 China; 2 Laboratory of Anesthesia and Critical Care Medicine, National-Local Joint Engineering Research Centre of Translational Medicine of Anesthesiology, West China Hospital, Sichuan University, Chengdu, Sichuan 610041 China; 3 Research Laboratory of Emergency Medicine, West China Hospital, Emergency Medicine and National Clinical Research Center for Geriatrics, Sichuan University, Chengdu, Sichuan 610041 China; 4 Department of Respiratory and Critical Care Medicine, Targeted Tracer Research and Development Laboratory, West China Hospital, Sichuan University, Chengdu, Sichuan 610041 China; 5 Department of Anesthesiology, New Jersey Medical School, Rutgers, The State University of New Jersey, Newark, NJ 07103 USA

**Keywords:** Memory impairment, Chronic neuropathic pain, Extracellular vesicles, microRNA, Dendritic spine

## Abstract

**Background:**

The pathogenesis of memory impairment, a common complication of chronic neuropathic pain (CNP), has not been fully elucidated. Schwann cell (SC)-derived extracellular vesicles (EVs) contribute to remote organ injury. Here, we showed that SC-EVs may mediate pathological communication between SCs and hippocampal neurons in the context of CNP.

**Methods:**

We used an adeno-associated virus harboring the SC-specific promoter Mpz and expressing the CD63-GFP gene to track SC-EVs transport. microRNA (miRNA) expression profiles of EVs and gain-of-function and loss-of-function regulatory experiments revealed that miR-142-5p was the main cargo of SC-EVs. Next, luciferase reporter gene and phenotyping experiments confirmed the direct targets of miR-142-5p.

**Results:**

The contents and granule sizes of plasma EVs were significantly greater in rats with chronic sciatic nerve constriction injury (CCI)than in sham rats. Administration of the EV biogenesis inhibitor GW4869 ameliorated memory impairment in CCI rats and reversed CCI-associated dendritic spine damage. Notably, during CCI stress, SC-EVs could be transferred into the brain through the circulation and accumulate in the hippocampal CA1-CA3 regions. miR-142-5p was the main cargo wrapped in SC-EVs and mediated the development of CCI-associated memory impairment. Furthermore, α-actinin-4 (ACTN4), ELAV-like protein 4 (ELAVL4) and ubiquitin-specific peptidase 9 X-linked (USP9X) were demonstrated to be important downstream target genes for miR-142-5p-mediated regulation of dendritic spine damage in hippocampal neurons from CCI rats.

**Conclusion:**

Together, these findings suggest that SCs-EVs and/or their cargo miR-142-5p may be potential therapeutic targets for memory impairment associated with CNP.

**Supplementary Information:**

The online version contains supplementary material available at 10.1186/s12974-024-03081-z.

## Introduction

Chronic neuropathic pain (CNP) is a common sequela of peripheral nerve injury and abnormal nervous system function [1]. With a prevalence ranging from 6.9 to 10% of the general population, CNP is a major contributor to the global disease burden [1, 2]. Substantial evidence from clinical and experimental research suggests that chronic pain often coexists with memory impairment [3, 4]. Peripheral nerve injury has been linked to abnormalities in short-term hippocampal working and recognition memory deficits and long-term potentiation deficits associated with spatial learning and memory disorders [5]. Persistent pain accelerates memory loss, affects daily activities, and decreases patient quality of life; for example, the relative risk of inability to manage finances independently is 11.8% greater [6]. Moreover, CNP has also been reported to induce genetic and structural changes in brain regions, including hippocampal volume reduction, abnormal hippocampal gliosis, and microtubule stability [3, 7–9]. Dendritic spines are tiny postsynaptic spines on the dendritic surface of neurons where synapses between neurons are formed. Changes in the number and morphology of spines are strongly correlated with learning and memory functions. Stress-induced dendritic spine remodeling in hippocampal neurons is somewhat reversible; however, prolonged and sustained stress may result in irreversible abnormal dendritic spine remodeling and impairment of hippocampal function, resulting in cognitive impairment. Recent studies have reported that the reduced number and density of dendritic spines in hippocampal neurons may be involved in the memory impairment associated with CNP [4]. However, current pain therapies are frequently ineffective for CNP. Moreover, neither effective nor ineffective of these therapies typically do not specifically target the rehabilitation of pain-related memory impairment [5]. Therefore, new strategies targeting the prevention or treatment of CNP-related memory impairment are urgently needed.

Schwann cells (SCs) are the most common glial cells in the peripheral nervous system and innervate peripheral nerves by interacting with axons and blood vessels [10]. SCs serve as a double-edged sword by engaging in axon creation and repair [11] but also perceiving harmful stimuli and relaying injurious information to nerves, triggering mechanical abnormalities in mouse models of neuropathic and cancer pain [10, 12]. Whether SCs participate in the onset of CNP-associated memory impairment is unknown.

Extracellular vesicles (EVs) are nanoscale membrane vesicles that are actively released by cells. They serve as important transduction mediators and mediate signal exchange between different cells and tissues [13]. Exosomes and microvesicles, which are derived from separate biogenesis routes and are referred to collectively as small EVs (sEVs), are two basic forms of EVs [14]. EVs are key mediators of protein and RNA transfer between glial cells and neurons and are involved in spatial transmission in the nervous system [15]. SC-derived EVs (SC-EVs) containing mRNA, miRNA, and protein cargoes are transferred into damaged axons and are crucial for axonal elongation and remyelination [11]. Little is known about whether SC-EVs can mediate communication between the sciatic nerve and the central nervous system, particularly in the exacerbation of brain pathology and memory impairment under CNP conditions.

In this study, we reported that concomitant memory impairment following chronic constriction injury (CCI) resulting from unilateral sciatic nerve injury was associated with reductions in dendritic spine density, dendritic complexity, and the expression of synapse-associated proteins in the hippocampus. Specifically, SC-EVs from injured sciatic nerves accumulated primarily in hippocampal CA1-CA3 cells through the bloodstream circulation. miR-142-5p was identified as the key molecule responsible for pathological SC-EVs-mediated memory impairment, and α-actinin-4 (ACTN4), ELAV-like protein 4 (ELAVL4, also known as HuD) and ubiquitin-specific peptidase 9 X-linked (USP9X) were identified as novel target molecules of miR-142-5p. ACTN4 is a class of actin-binding proteins whose primary function is to cross-link actin filaments into bundles. Crosslinking of actin filaments provides rigidity and stability for filaments. ACTN4 Governs Dendritic Spine Dynamics [16]. ELAVL4 is a member of the human antigen/ELAV-like family of RNA-binding proteins (RBPs) that are mostly expressed in neurons but also in the pancreas and testis at lesser levels [17]. ELAVL4 is essential for proper neural development, nerve regeneration, and synaptic plasticity, and its dysregulation is involved in several pathologies [18, 19]. USP9X is a neurodevelopmental-disorder-associated deubiquitinase and involve in dendritic spine development [20]. Reducing EV secretion, interfering with miR-142-5p expression or overexpressing ACTN4, ELAVL4 and USP9X ameliorated memory impairment and rescued CNP-associated dendritic spine changes. Together, these findings provide reliable evidence that SC-EVs mediate pathologic communication between dysfunctional sciatic nerves and the brain.

## Materials and methods

### Animals

A total of roughly 230 Male SD rats (5–8 weeks, 180–220 g) were purchased from Chengdu Dashuo Laboratory Animal Co. Rats were housed under controlled conditions (SPF) with free access to food and water and a 12-hour light/dark cycle.

### Cell culture

HT-22 and HEK293T cells (Huiying Biological Technology Co., Ltd., Shanghai, China) were maintained in Dulbecco’s modified Eagle’s medium (DMEM) supplemented with 4.5 g/L glucose, 10% fetal bovine serum, 1% penicillin‒streptomycin and 5% CO_2_. The isolated embryonic hippocampal neurons were cultured as previously described [21]. Briefly, pregnant rats aged 15 and 17 days were anesthetized, and the hippocampi of the unborn rats were isolated under a dissection microscope at 4 °C. The hippocampal tissue was sliced into tiny pieces using scissors and digested for 8–12 min with 0.25% trypsin. After trituration with the plating media, the neurons were filtered through a 40 μm cell sieve and plated onto a cover slip or a 6-well cell culture plate coated with poly-D-lysine. The neurons were incubated in a humidified incubator at 37 °C with 5% CO_2_ for 10 min before the media was changed to neurobasal medium supplemented with 2% B27.

### CNP model establishment and treatment

Rat models of CNP induced by peripheral nerve injury were established as described previously [22, 23]. The details of the CNP rat model are provided in the Supplementary Methods section. We successfully constructed rat CNP models using CCI, partial sciatic nerve ligation (PSNL) and spared nerve injury (SNI), respectively(Supplementary Fig. 1 and Supplementary Fig. 2). To inhibit exosome synthesis, the neutral sphingomyelinase-2 (nSMase2) inhibitor GW4869 (2 mg/kg) was intraperitoneally administered three times per week for three weeks in vivo [24].

### Behavioral tests

Behavioral tests (including the PWT, PWL test, acetone test, Y maze test and object recognition test) were carried out by two trained operators who were blinded to the group to which the animals belonged. Rats were habituated to the testing environment for 2 h before testing. Specific information can be found in the Supplementary Methods section.

### AAV vector construction, production, titration, and injection

AAV vector construction, production and titration were performed as described in the Supplementary Methods section. In brief, rats were anesthetized with 2-3% isoflurane, and the sciatic nerve and dorsal root ganglion (DRG) were exposed as described in previous studies [25, 26]. A volume of 4 µL of the viral vectors was manually and gently injected into the sciatic nerve/DRG through a 33-gauge needle (Hamilton syringe). The needle was kept at the injection location for 1 min before being carefully withdrawn.

### Golgi and DIL/DIO staining

Golgi staining was performed utilizing the FD Rapid Golgi StainTM Kit according to the manufacturer’s instructions (FD NeuroTechnologies, USA). Briefly, rat brains were washed in distilled water and impregnated with an impregnation solution for 14 days. The slices were sliced to 150 μm thickness with a vibratome (Leica, Wetzlar, Germany), mounted on slides, and dyed with chemicals from the kit. A Zeiss LSM 710 Duo microscope with 20X lenses was used to acquire Z-stack images of the neurons. With 100X objectives, dendritic spines were collected. Sholl analysis was performed with ImageJ to assess the total dendritic length, number of branch points, and spine density. With ImageJ software, each spine head was manually specified in the channel of the cell fill to pinpoint the location. Dendritic spines were classified as previously reported [27]. Specifically, the criteria for spine classification were as follows: spines with lengths greater than 3 m and less than 10 m were classified as “filopodia,” spines with lengths longer than widths were classified as “long, thin,” spines with lengths shorter than widths were classified as “stubby,” and spines with head widths longer than the neck width were classified as “mushroom.” The filopodia and long thin pseudopods are immature, while the stubby and mushroom forms are adults. For DIL/DIO staining, cells were fixed with 2% paraformaldehyde for 15 min at room temperature. Then, 5 µM DIL (Beyotime Biotechnology, Cat No. C1036) or DIO (Beyotime Biotechnology, Cat No. C1038) was added to the cells, which were incubated for 15 min at RT. Before imaging, the cells were washed twice with PBS. Images were acquired using an autoinverted fluorescence microscope (Olympus, Japan) with a 100X objective.

### Transfection of primary neurons with AAV, miRNA agomir or antagomir

For AAV transduction in primary neurons, the viral particles were added to neurobasal medium at a multiplicity of infection of 1 × 10^6^ on the second day of plating. The neurons were analyzed on 14 days in vitro (DIV). The transduction efficiency was observed at > 90%. For miRNA transduction in primary neurons, the neurons were transfected on DIV 12. According to the manufacturer’s instructions, Lipo3000 (L3000008, Invitrogen) was used to transfect 100 nm of miRNA agomir or antagomir into each well of cells using Opti-MEMTM I Reduced Serum Medium (31985062, Thermo Fisher). The cells were examined 48 h after miRNA agomir or antagomir transfection. The miRNA agomir, antagomir, negative control NC agomir, and NC antagomir were purchased from RiboBio (Guangzhou, China).

### miRNA agomir and antagomir injection

Rats were anesthetized with 2-3% isoflurane. For bilateral hippocampal stereotactic injection, the miR-142-5p agomir was injected into the hippocampus of rats using the following coordinates: anteroposterior, ± 4.3 mm; medial and lateral, ± 2.8 mm; and dorsoventral, -2 mm. After 48 h, brain tissues were harvested for further experiments. For injection of the miRNA agomir into the sciatic nerve, the miR-142-5p antagomir (2 nmol/rat) was injected into the sciatic nerve once a week for 3 weeks after CCI induction.

### Dual-luciferase reporter assay

HEK293 cells were plated on a 96-well plate (2 × 103 cells/well) overnight. Using the riboFECT CP Transfection Kit (Ruibo, C10511-05), the pmiR-RB-ReportTM vector containing the WT or mutant 3′UTRs of ACTN4 (MUT), ELAVL4 (MUT1 and MUT2), or USP9X (MUT) was cotransfected into HEK293T cells with 100 nm agomir miRNA or agomir control. According to the manufacturer’s protocol, cell lysates were collected after 48 h. The activities of firefly and Renilla luciferases were detected by the Dual Luciferase Reporter Assay System (Vazyme, DL101-01). The absorbance values were read using a Synergy™ HTX Multi-Mode Microplate Reader (BioTek Instruments).

### Separation of extracellular vesicles from tissue

EVs were isolated from tissue using a protocol developed previously by Vella et al., with minor modifications [28]. The dissociation mixture was formulated utilizing the Miltenyi Human Tumor Dissociation Kit (Miltenyi Biotec, cat. no. 130-095-929). The details are listed in the Supplementary Methods section.

### Cellular uptake of labeled exosomes from rat plasma

Exosomes from the plasma of normal and model rats were labeled with 1,1′-dioctadecyl-3,3,3′,3′-tetramethylindocarbocyanine perchlorate (DiI) fluorescent dye (Beyotime Biotechnology, C1036). Briefly, the EV solution was incubated with Dil (1 µmol) for 10 min in the dark at room temperature (RT), followed by a single wash with PBS and ultracentrifugation (100,000 × g) for 1 h at 4 °C. HT22 cells were incubated with DiI-labeled exosomes for 3 h (cell-to-EV ratio of 1:300). The cells were fixed with 4% paraformaldehyde. The cells were permeabilized with 0.1% Triton X-100 in PBS for 10 min and then blocked with 5% bovine serum albumin at room temperature for 1 h. Subsequently, the cells were stained with FITC-phalloidin (Beyotime Biotechnology, C1003) for 30 min at RT in the dark. Nuclei were stained with DAPI. A fluorescence microscope (Olympus, IX83, Japan) was used for observation and imaging.

### NTA, TEM, microbead-assisted flow cytometry, and other experiments

NTA, TEM, SEM, microbead-assisted flow cytometry, immunofluorescence, western blot, RT‒qPCR, library preparation, sequencing, and quantification and differential expression analysis of miRNAs were conducted as described in the Supplementary Information: Supplementary Methods.

### Statistical analysis

GraphPad Prism 9.0 was used for statistical analysis. For comparisons between two groups, unpaired tests or two-way analysis of variance (ANOVA) tests with Šídák’s multiple comparisons test were used. Statistical differences among the 3 groups were determined using two-way ANOVA with Tukey’s multiple comparisons test. All the data are presented as the means ± SDs. A p value < 0.05 was considered to indicate statistical significance.

## Results

### Plasma_EV^CCI^ exacerbates memory impairment and dendritic spine damage

**Fig. 1:**
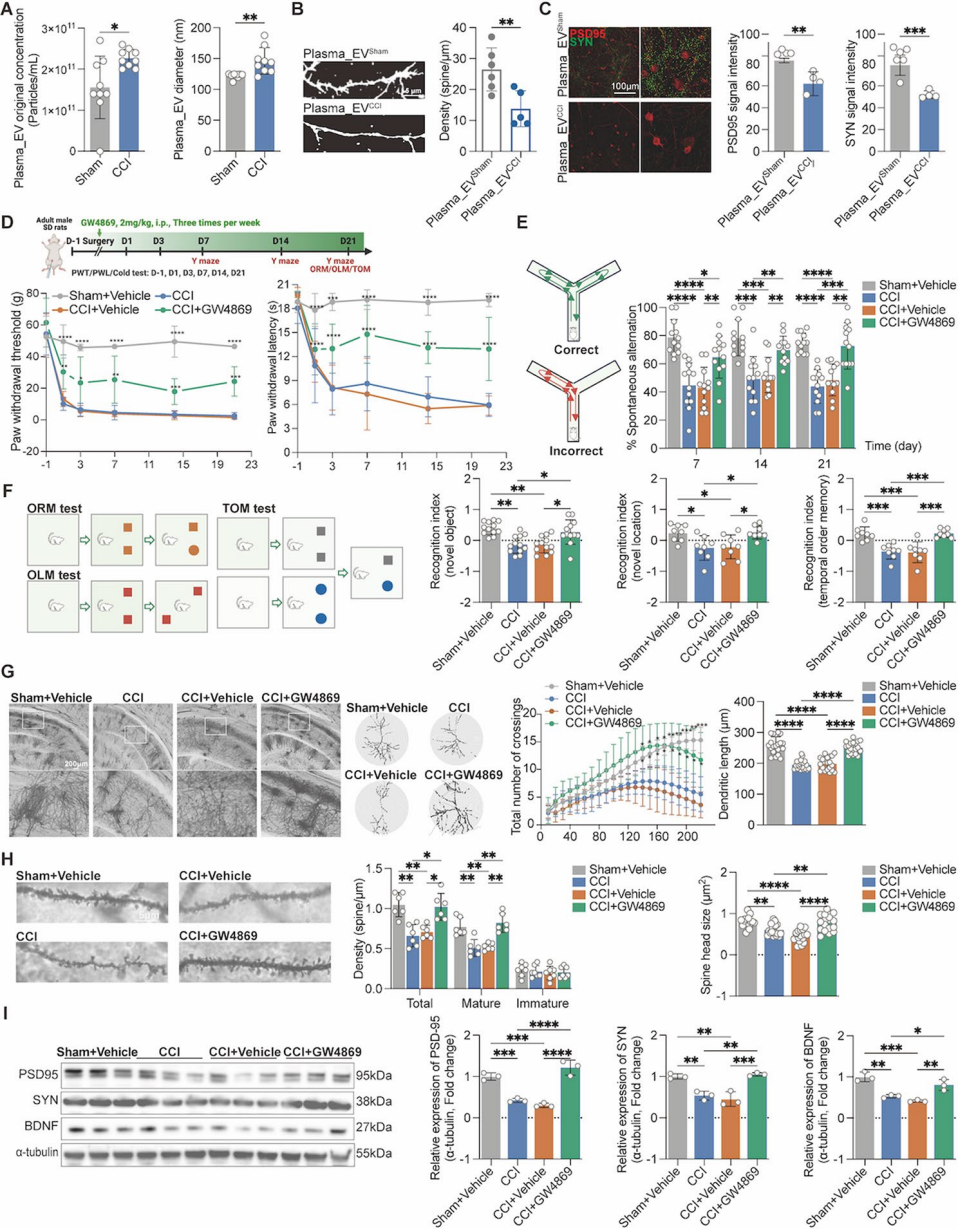
Plasma_EV^CCI^ damages neuronal dendritic spines, and the EV inhibitor GW4869 alleviates hyperalgesia and memory impairment in CCI rats. Plasma EVs were extracted by differential high-speed centrifugation of the rats’ plasma. (**A**) NTA analysis showed that the concentration and diameter of plasma EVs were greater in CCI rats than in sham rats. *n* = 9 rats. unpaired Student’s t test was used. (**B**, **C**) DIL staining and immunofluorescence staining showed that plasma_EV^CCI^  significantly inhibited the dendritic spine density and the expression levels of PSD95 and SYN proteins in primary hippocampal neurons. *n* = 4–6 images from three wells of each group. unpaired Student’s t test was used. (**D**) Flowchart for establishing the CCI model, GW4869 intervention, evaluation of nociceptive hypersensitivity and memory behavior evaluation; The paw withdrawal threshold (PWT), and paw withdrawal latency (PWL) were each determined using Von Frey filaments and thermal pain stimulators, respectively. GW4869 treatment substantially raised the PWT and PWL at 1, 3, 7, 14 and 21 days after CCI. *n* = 12 rats, two-way ANOVA followed by Tukey’s multiple comparisons test was used. (**E**) Compared with CCI or vehicle treatment, GW4869 treatment enhanced the spontaneous alternation behavior of CCI rats. *n* = 10 rats. two-way ANOVA followed by Tukey’s multiple comparisons test was used. (**F**) Compared with CCI or vehicle treatment, GW4869 treatment increased the recognition indices of CCI rats in the ORM, OLM and TOM tests. *n* = 8–10 rats. two-way ANOVA followed by Tukey’s multiple comparisons test was used. (**G**, **H**) After Golgi staining, Sholl analysis and ImageJ analysis showed that GW4869 rescued the dendritic complexity, dendritic length, density, and spine head size in hippocampal neurons from CCI rats compared with neurons from the hippocampus of CCI or CCI + Vehicle rats. *n* = 5–6 stained sections from 3 animals in each group. ordinary one-way followed by Tukey’s multiple comparisons test was used for dendritic length; mixed-effects analysis followed by Tukey’s multiple comparisons test was used for dendritic density and spine head size; two-way ANOVA followed by Tukey’s multiple comparisons test was used for dendritic complexity (**I**) GW4869 improved PSD95, SYN and BDNF protein levels in the hippocampus of CCI rats. The data are shown as the means ± SDs. ordinary one-way followed by Tukey’s multiple comparisons test was used. *P < 0.05, **P < 0.01, ***P < 0.001, ****P < 0.0001

We first collected EVs from the plasma of rats in the sham and CCI groups by differential ultracentrifugation and characterized them by transmission electron microscopy (TEM). The particle size of the extracted EVs was mainly distributed between 50 and 300 nm, with typical vesicular structures (Supplementary Fig. 3A). The expression of the marker proteins CD9, TSG101 and HSP70 in these EVs was detected (Supplementary Fig. 3B). Moreover, nanoparticle tracking analysis (NTA) revealed that the particle size and concentration of plasma EVs were significantly greater in the CCI group than in the sham group 14 days after surgery (Fig. 1A and Supplementary Fig. 3C). To investigate the influence of plasma EVs on hippocampal dendritic spines, we performed DIL staining following a 24-hour treatment of primary hippocampal neurons with plasma_EV^sham^ and plasma_EV^CCI^. The plasma_EV^CCI^ reduced the number of dendritic spines in primary hippocampal neurons (Fig. 1B). In addition, the expression levels of the neuronal synapse-associated proteins PSD95 and SYN were decreased after treatment of primary hippocampal neurons with plasma_EV^CCI^ (Fig. 1C). To further determine the role of plasma EVs in CCI-associated memory impairments, we subcutaneously injected the classical EV inhibitor GW4869 into rats and assessed their memory behaviors after CCI (Fig. 1D). Unexpectedly, GW4869 significantly increased PWT and PWL at 1, 3, 7, 14 and 21 days after CCI (Fig. 1D). Furthermore, GW4869 substantially enhanced spontaneous alternation behaviors in CCI rats in the Y maze test at 7, 14, and 21 days after CCI (Fig. 1E), as well as improving the memory recognition indices of CCI rats 21 days after CCI in the ORM, OLM, and TOM tests (Fig. 1F). Moreover, the PWT, PWL, and cold pain score/duration in the CCI rats were also improved after GW4869 intervention (Supplementary Fig. 3D). Furthermore, we examined the changes in hippocampal neuronal dendritic spines and found that hippocampal (CA1 and CA2) neuronal dendritic spine density, dendritic length, total/mature dendritic spine density and spine head size were significantly lower in both CCI rats and CCI + Vehicle rats; however, the EV inhibitor GW4869 significantly reversed the above pathological changes in CCI rats (Fig. 1G–I).

### Schwann cell-derived EVs migrate to the blood circulation to accumulate in the hippocampus

**Fig. 2:**
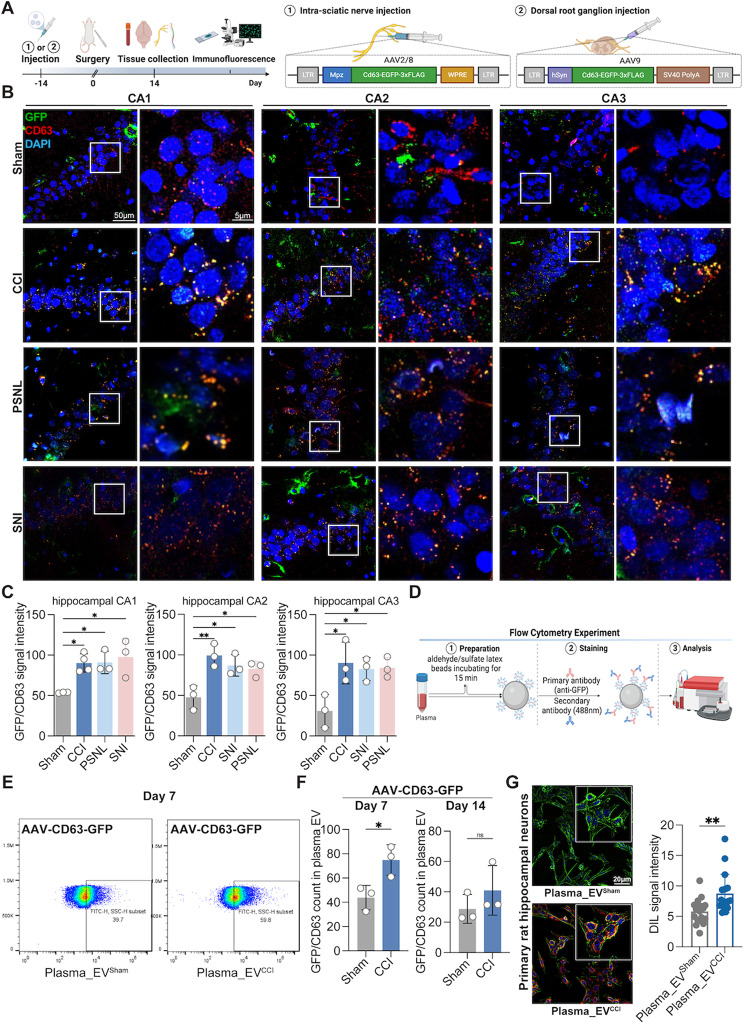
Tracing of specifically labeled Schwann cell-derived EVs in the hippocampus and plasma. (**A**) AAV design, construction, and flowchart for AAV injection. (**B**, **C**) Compared to those in the sham group, GFP-CD63 signal intensity (Meaning Schwann cells secreting GFP-CD63-specific labeled EVs) were significantly clustered in the hippocampal CA, CA2 and CA3 regions of CNP model rats with memory impairment, including the CCI, PSNL and SNI models. *n* = 3–4 rats. ordinary one-way followed by Dunnett’s multiple comparisons test was used. (**D**) Protocol for microbead-assisted flow cytometry. (**E**, **F**) Microbead-assisted flow cytometry revealed that the GFP/CD63 labeled-cell ratio in the plasma EVs of CCI rats was greater than that in those of sham rats on day 7 after modeling. There was no change in the GFP/CD63 ratio between the two groups on day 14 postmodeling. *n* = 3 rats. unpaired Student’s t test was used. (**G**) Images of primary rat hippocampal neurons co-incubated with sham or CCI rat plasma EVs after labeling with DIL revealed increased plasma_EV^CCI^ within the neurons. *n* = 3–4 images from three wells of each group. unpaired Student’s t test was used. The data are shown as the means ± SDs. ns, no significant difference; *P < 0.05; **P < 0.01

To verify the role of SC-EVs in the central nervous system (CNS) under CNP stress, we designed and constructed an AAV2/8 viral vector containing the Schwann cell-specific promoter Mpz (AAV-Mpz-CD63-GFP) and an AAV9 viral vector containing the neuron-specific promoter hSyn (AAV-hSyn-CD63-GFP) (Fig. 2A). To verify the specificity of the viral vector transduction, we injected the AAV viruses described above into the sciatic nerves and DRGs of the rats and collected the sciatic nerves and DRG tissues 14 days later (Fig. 2A). After staining the sciatic nerve and DRG with DAPI, GFP, and S100β/MAP2, strong fluorescent signals of GFP (colocalized with S100β/MAP2) were observed in the sciatic nerve and DRG (Supplementary Fig. 3E, F), indicating that the specific cell-expressing viral vector had improved transduction efficiency and cell specificity.

We subsequently investigated whether SC-EVs exposed to CNP stress could enter the central nervous system and impact memory function. AAV-Mpz-CD63-GFP, AAV-hSyn-CD63-GFP, and the control were administered through injection into the sciatic nerve of rats to introduce CD63-GFP into SC-EVs. The experimental model was then established after 14 days. The brain tissues were harvested after 14 days of modeling and subsequently subjected to co-staining using DAPI, GFP, and CD63. Our results showed that after AAV-Mpz-CD63-GFP transduction, CCI rats exhibited significant CD63-GFP signals, primarily in the hippocampal CA1/2/3 area (Fig. 2B, C), with a minor amount of signal in the hippocampal DG area (Supplementary Fig. 4A). In contrast, no CD63-GFP signals were present in the brains of the rats injected with the control virus, indicating that EVs released from SCs could reach the CNS and enrich the hippocampus. There was little CD63-GFP signal in the cortex and prefrontal cortex of the rats in both groups (Supplementary Fig. 5A). In addition, we observed that hippocampal neurons exhibited a significant increase in CD63-GFP signals, while astrocytes and microglia exhibited a conspicuous lack of CD63-GFP signals (Supplementary Fig. 5B–D). Moreover, we pondered whether this occurrence was duplicated in alternative CNP models. The results showed that the CD63-GFP signals in the hippocampal CA1/2/3 region significantly increased in the PSNL and SNI rat models transfected with AAV-Mpz-CD63-GFP (Fig. 2B, C).

Furthermore, we injected AAV-hSyn-CD63-GFP into the rat DRG and evaluated the enrichment of EVs in the hippocampus and prefrontal cortex on day 14 following CCI, and we found almost no CD63-GFP signals in the hippocampus, cortex, or prefrontal cortex in either group (Supplementary Fig. 6A–F). Therefore, our subsequent experiments focused on SC-EVs. We further sought to investigate the potential correlation between the concentration of SC-EVs in the hippocampus and the exact time point at which samples were collected for the purpose of modeling. After successful transfection of rats with AAV-Mpz-CD63-GFP, a model was established. After the experimental procedure, entire brains were collected at 3, 7, and 21 days after the modeling process. At the aforementioned time points, some CD63-GFP signals were observed in the hippocampal CA1/2/3 region of the CCI group, but only a tiny quantity of signals was observed in the hippocampus of the sham group (Supplementary Fig. 7A- C). Furthermore, our results showed a significant increase in fluorescein sodium leakage within the hippocampal region in rats exposed to CCI compared to sham rats (Supplementary Fig. 8A). This observation suggested increased permeability of the blood‒brain barrier and damage to the blood‒brain barrier in CCI rats. Interestingly, no significant difference was observed in the cortical region (Supplementary Fig. 8A).

To further understand the source of SC-EV accumulation in the hippocampus, we investigated possible pathways involved in axoplasmic transport and circulation. First, we collected sciatic nerve, DRG, and spinal cord tissues from rats after AAV-Mpz-CD63-GFP transduction and evaluated the CD63-GFP signals using laser confocal microscopy. We discovered that CD63-GFP signals were significantly upregulated in the sciatic nerve (Supplementary Fig. 4B). Nevertheless, few CD63-GFP signals were observed in the DRG and spinal cord across all the experimental groups (Supplementary Fig. 4C, D), indicating that SC-EVs may not reach the CNS via the spinal cord pathway. EVs are rarely examined using flow cytometry because their nanoscale size surpasses the detection limit of flow cytometry. We used microbead-aided flow cytometry to detect GFP-labeled EVs in plasma according to previous reports [24] (Fig. 2D). Both transmission electron microscopy (TEM) and scanning electron microscopy (SEM) indicated that the collected EVs were densely coated on the microbeads, indicating EV enrichment on the beads (Supplementary Fig. 8B). Following AAV-Mpz-CD63-GFP virus transduction, we isolated EVs from the plasma of the rats (at 7 and 14 days postmodeling) (Fig. 2D). Furthermore, GFP-labeled EVs were significantly more abundant in the plasma of CCI rats (7 days after modeling) than in that of sham rats (Fig. 2E, F). On day 14 of modeling, there was a trend toward a difference between the two groups, but there was no significant difference (Fig. 2F). Furthermore, after treating primary hippocampal neurons with plasma_EV^sham^ or plasma_EV^CCI^ for 24 h, we discovered that they were predominantly rich in plasma_EV^CCI^, as indicated by immunofluorescence staining (Fig. 2G).

### Identification of miR-142-5p as the main carrier of SC-EVs^CCI^ enriched in the hippocampus

Although EVs contain a range of signaling molecules involved in cell‒cell communication, there is growing evidence that miRNAs are essential molecules in the regulation of receptor cell activity by EVs [29]. As a result, we hypothesized that miRNAs transported by SC-EVs were engaged in dendritic spine remodeling in CCI-associated memory impairment.

**Fig. 3:**
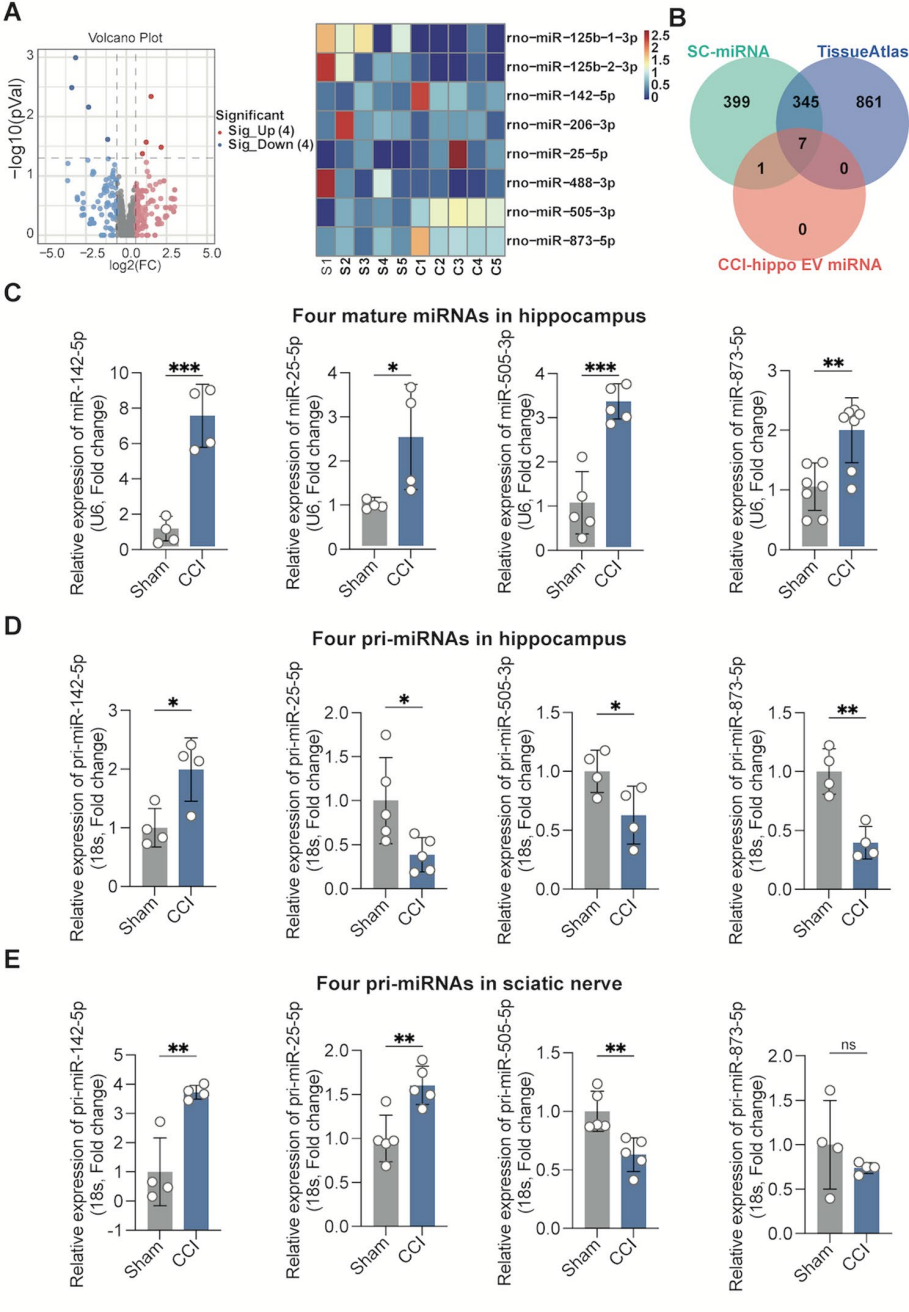
miR-142-5p was increased in the hippocampus of rats in the CCI-associated memory impairment model. (**A**) Differentially expressed miRNAs were selected from sequencing data of hippocampal EV miRNAs. (**B**) Venn analysis of the seven candidate miRNAs satisfying the three conditions. (**C**) RT‒qPCR showed that the relative expression of mature miR-142-5p, mature miR-25-5p, mature miR-505-3p, and mature miR-873-5p were obviously upregulated in the hippocampus of CCI rats compared with that in sham rats. *n* = 4–6 rats. unpaired Student’s t test was used. (**D**) RT‒qPCR results showing that the relative expression of pri-miR-25-5p, pri-miR-505-3p and pri-miR-873-5p were downregulated in the hippocampus of CCI rats, but that of pri-miR-142-5p was upregulated compared with that in sham rats. *n* = 4–5 rats. unpaired Student’s t test was used. (**E**) RT‒qPCR results showing that the relative expression of mature miR-142-5p and miR-25-5p were increased in the sciatic nerves of CCI rats, but that the expression of mature miR-505-3p was decreased; moreover, there was no difference in the expression of mature miR-505-3p compared with that in sham rats. *n* = 4–6 rats. unpaired Student’s t test was used. The data are shown as the means ± SDs. ns, no significant difference; *P < 0.05; **P < 0.01

Five series of procedures were conducted. Initially, hippocampal tissues were harvested from the rats in the sham or CCI group for 14 days and then centrifuged to extract hippocampal EVs. The whole hippocampi from three rats per group were mixed, and EVs were isolated. EV-containing small RNAs were isolated and subjected to deep RNA-seq analysis (each sample included genetic information from 3 rats). Global miRNA profiling revealed significant upregulation of 4 miRNAs and downregulation of 4 miRNAs in hippocampal EVs (filtering criteria, *p* < 0.05, |log 2(FC)| > = 0.58) (Fig. 3A). Subsequently, to predict that EV-containing miRNAs potentially promote dendritic spine remodeling, miRNAs significantly increased in hippocampal EVs were analyzed against three miRNA databases: Schwann-enriched miRNAs [30], the Tissue Atlas database (https://ccb-web.cs.uni-saarland.de/tissueatlas/hsa_vs_rno), and the Sham/CCI rats’ hippocampus differential miRNAs via our RNAseq. Venn overlap analysis revealed 7 common miRNAs, including 4 significantly upregulated miRNAs (Fig. 3B).

Furthermore, to evaluate whether mature hippocampal miRNAs (the aforementioned increased miRNAs) were of sciatic nerve origin, primary (pri) miRNAs were detected simultaneously in the hippocampus and sciatic nerve tissue by real-time qPCR. Then, a miDETECT A Track miRNA qRT‒PCR starter kit was used to determine miRNA expression in the hippocampus. The expression of miR-142-5p was most notably increased in the hippocampus (> 7-fold), and the expression of miR-25-5p (> 3-fold), miR-505-3p (> 3-fold), and miR-873-5p (> 3-fold) were modestly increased (Fig. 3C). In addition, an increase in pri-miR-142-5p was observed in the hippocampus, but the expression of pri-miR-25-5p, pri-miR-505-3p, and pri-miR-873-5p were obviously downregulated in the hippocampus (Fig. 3D). In addition, pri-miR-142-5p and pri-miR-25-5p were highly expressed in the sciatic nerve, with a 3-fold increase in pri-miR-142-5p (Fig. 3E). Interestingly, primary miR-505-3p was markedly downregulated in the sciatic nerve, whereas pri-miR-873 was unchanged in the sciatic nerve. These results provide clear evidence that miR-142-5p and miR-25-5p may be produced by injured sciatic nerves and translocated to the hippocampus via EVs. Several studies have indicated that miR-142-5p plays a role in memory impairment in models of Alzheimer’s disease, PTSD, and traumatic brain injury [31–33]. We subsequently selected miR-142-5p, which exhibited the greatest difference in expression, for our study.

### miR-142-5p mediates memory impairment and hippocampal neuronal dendritic spine damage via SC-EVs transport

**Fig. 4:**
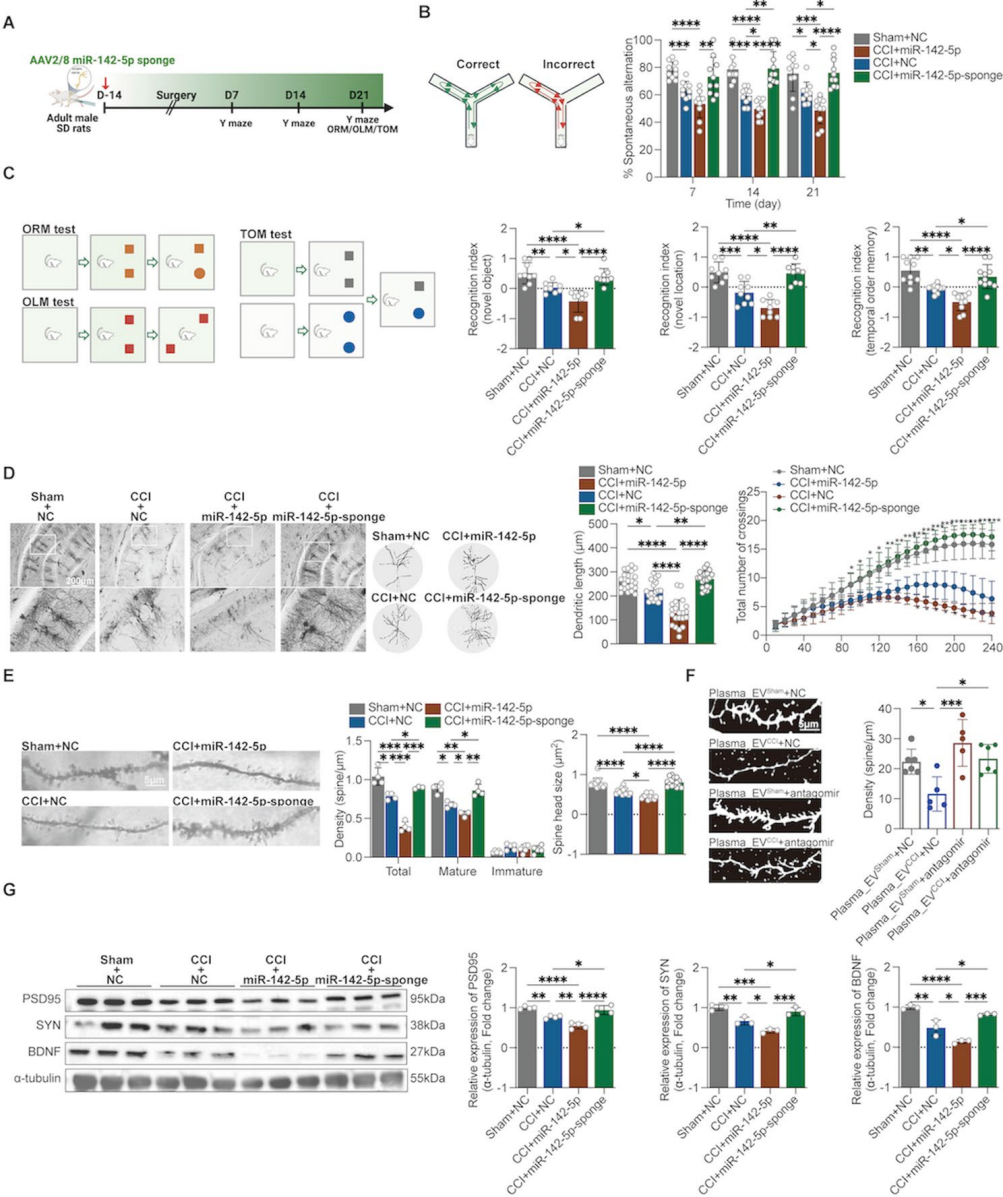
Identification of miR-142-5p as a molecule responsible for SC-EV-induced memory impairment and dendritic spine damage. We designed and constructed an AAV carrying a specific Schwann cell promoter that modulates (upregulates or encloses) miR-142-5p expression in Schwann cells after sciatic nerve injection. (**A**) Flowchart for establishing the CCI model, injecting AAV, and evaluating memory-related behaviors. (**B**) The AAV-miRNA-142-5p sponge enhanced the spontaneous alternation behavior of CCI rats compared with that of rats in the CCI + AAV-NC group; however, AAV-miR-142-5p aggravated this behavior. *n* = 9–10 rats. two-way ANOVA followed by Tukey’s multiple comparisons test was used. (**C**) The AAV-miRNA-142-5p sponge increased the recognition index of CCI rats in the ORM, OLM and TOM tests compared with that of rats in the CCI + AAV-NC group; however, AAV-miR-142-5p aggravated this index. *n* = 8–10 rats. ordinary one-way followed by Tukey’s multiple comparisons test was used for OLM and TOM; mixed-effect analysis followed by Holm-Šídák’s multiple comparisons test was used for ORM; (**D**, **E**) After Golgi staining, Sholl analysis and ImageJ analysis showed that, compared with those in neurons in the rat hippocampus of the CCI + AAV-NC group, the AAV-miRNA-142-5p sponge rescued the dendritic complexity, dendritic length, density, and spine head size in hippocampal neurons of CCI rats, but AAV-miR-142-5p exacerbated these parameters. *n* = 5–6 stained sections from 3 animals in each group. ordinary one-way followed by Tukey’s multiple comparisons test was used for spine head size and dendritic length; two-way ANOVA followed by Tukey’s multiple comparisons test was used for dendritic complexity; mixed-effects analysis followed by Tukey’s multiple comparisons test was used for dendritic density; (**F**) DIL staining showed that the miR-142-5p antagomir effectively counteracted the reduction in dendritic spine density observed in primary hippocampal neurons following exposure to plasma_EV^CCI^. *n* = 6 images from three wells per group. ordinary one-way followed by Tukey’s multiple comparisons test was used. (**G**) The AAV-miRNA-142-5p sponge increased PSD95, SYN and BDNF protein levels in the hippocampus of CCI rats, but AAV-miR-142-5p decreased PSD95, SYN and BDNF protein levels. Ordinary one-way followed by Tukey’s multiple comparisons test was used. The data are shown as the means ± SDs. *P < 0.05, **P < 0.01, ***P < 0.001, ****P < 0.0001

Therefore, we performed gain-of-function and loss-of-function experiments to obtain direct evidence of miR-142-5p-induced memory impairment and dendritic spine damage in Schwann cells of the sciatic nerve. We designed and constructed AAV viruses containing the specific Schwann cell promoter Mpz, AAV-miR-142-5p sponge and AAV-miR-142-5p to block or upregulate miR-142-5p expression in Schwann cells by sciatic nerve injection for 14 days after modeling, after which cognitive behavioral tests were performed (Fig. 4A). The results showed that the AAV-miR-142-5p sponge improved the spontaneous alternation of CCI rats at 7, 14 and 21 days in the Y-maze and improved the cognitive recognition indices of CCI rats at 21 days in the novel object recognition, location recognition, and temporal sequencing experiments; however, the AAV-miR-142-5p aggravated the memory impairment of the CCI (Fig. 4B, C). In addition, we assessed pain and memory behaviors after sciatic nerve injection of the miR-142-5p antagomir (Supplementary Fig. 8C). Additionally, sciatic nerve injection of the miR-142-5p antagomir improved spontaneous alternating behaviors in CCI rats in the Y maze on days 7, 14, and 21 postmodeling; improved memory recognition indices in the ORM, OLM, and TOM experiments on day 21 postmodeling; and improved the PWT, PWL, and cold pain scores/durations in CCI rats (Supplementary Fig. 8D, E).

Furthermore, we found that the AAV-miR-142-5p sponge improved hippocampal (CA1 and CA2) dendritic length, dendritic spine density, the number of mature dendritic spines, spine head size, as determined by Golgi staining and the expression levels of the synapse-associated proteins PSD95, SYN, and BDNF in CCI rats (Fig. 4D, E, G). AAV-miR-142-5p aggravated the above pathological changes in CCI rats. Additionally, our study revealed that the administration of the miR-142-5p antagomir effectively counteracted the reduction in dendritic spine density observed in primary hippocampal neurons following exposure to plasma_EV^CCI^ via DIL staining (Fig. 4F). These findings point to the involvement of sciatic nerve-derived miR-142-5p in the onset of CCI-mediated memory impairment.

### Identification of ACTN4, ELAVL4 and USP9X as important targets of miR-142-5p

**Fig. 5:**
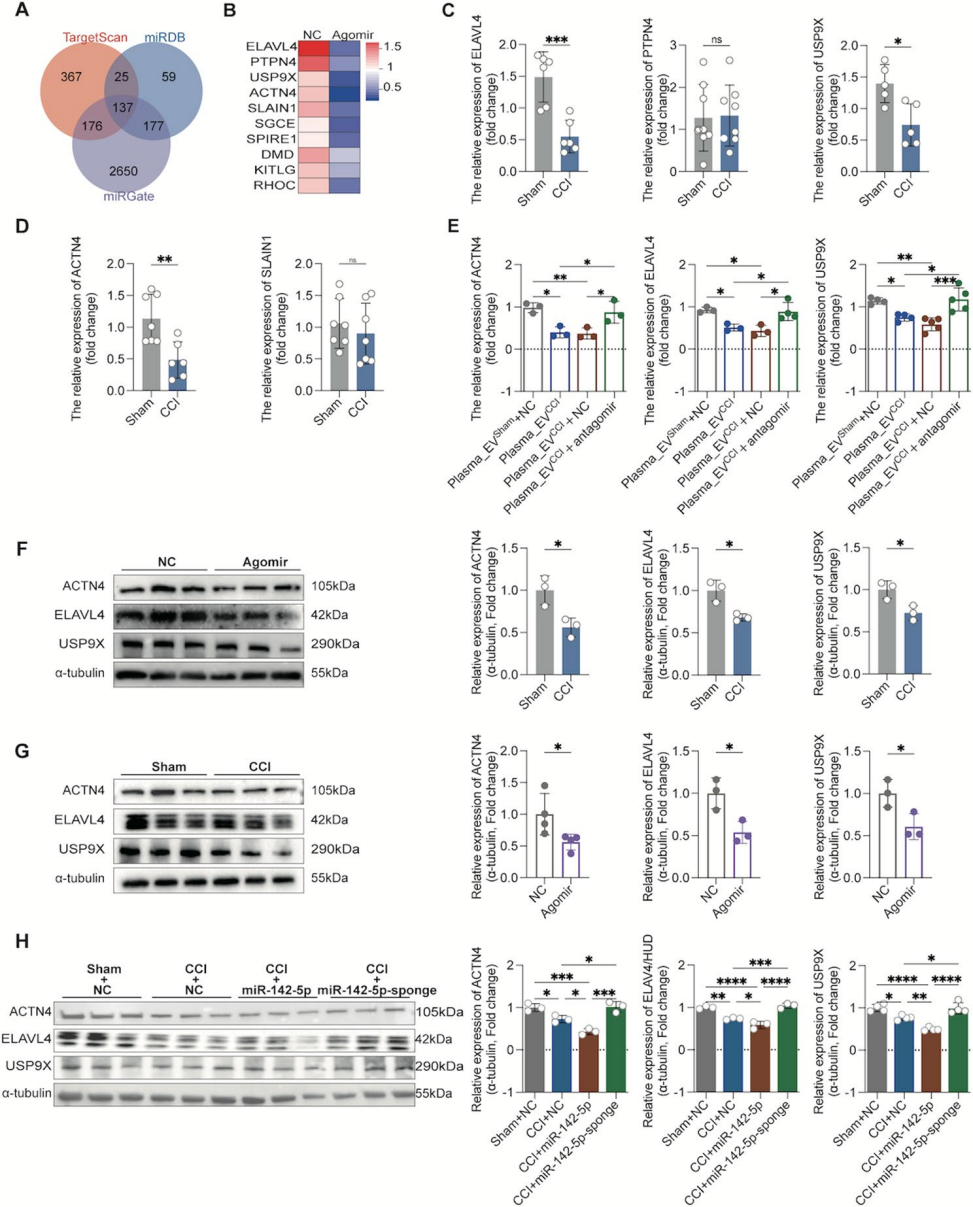
ACTN4, ELAVL4 and USP9X are direct targets of miR-142-5p and contribute to miR-142-5p-induced memory impairment and dendritic spine damage. (**A**) miR-142-5p was predicted using TargetScan, miRDB and miRGate, and the predicted data were subsequently subjected to Venn analysis. (**B**) Intersecting genes were subjected to GO pathway enrichment analysis, and 10 genes in the dendritic spine-associated actin pathway were selected for validation. miR-142-5p agomir and NC were injected into the normal rat bilateral hippocampus via stereotaxic localization. After 48 h, RT-qPCR showed that the mRNA levels of ELAVL4, PTPN4, USP9X, ACTN4, and SLAIN1 in the hippocampal tissues of the rats in the miR-142-5p agomir intervention group were significantly lower, while the other genes were not significantly different. *n* = 3–6 rats. unpaired Student’s t test was used. (**C**, **D**) RT‒qPCR revealed that ACTN4, ELAVL4 and USP9X mRNA levels were significantly lower in the hippocampal tissues of CCI rats than in those of sham rats. *n* = 5–8 rats. unpaired Student’s t test was used. (**E**) RT‒qPCR revealed that the miR-142-5p antagomir significantly reversed the inhibitory effect of plasma_EV^CCI^ on the mRNA levels of ACTN4, ELAVL4 and USP9X in primary hippocampal neurons. ordinary one-way followed by Tukey’s multiple comparisons test was used. (**F**) Western blot analysis showed that the protein levels of ACTN4, ELAVL4 and USP9X were significantly lower in the hippocampal tissues of rats stereotaxically injected with the miR-142-5p agomir than in those of the NC group. *n* = 3 rats. unpaired Student’s t test was used. (**G**) Western blot analysis revealed that ACTN4, ELAVL4 and USP9X protein levels were significantly lower in the hippocampal tissues of CCI rats than in those of sham rats. *n* = 3–4 rats. unpaired Student’s t test was used. (**H**) AAV-miR-142-5p sponge significantly improved the decreased ACTN4, ELAVL4 and USP9X protein levels in hippocampal tissues of CCI rats compared to the CCI + AAV-NC group; however, AAV-miR-142-5p exacerbated the decreased protein expression, via Western blot analysis. *n* = 3–4 rats. ordinary one-way followed by Tukey’s multiple comparisons test was used. The data are shown as the means ± SDs. *P < 0.05, ** *P* < 0.01, ***P < 0.001, ****P < 0.0001

Bioinformatics analyses and experimental validation were used to identify miR-142-5p target genes that are objectively responsible for dendritic spine remodeling. We analyzed the potential target genes of miR-142-5p by prediction via the TargetScan, miRDB and miRGate databases, and 137 target genes were identified after intersection analysis via Venn analysis (Fig. 5A). The 137 target genes were subjected to GO enrichment analysis using DAVID tools, and the enrichment pathway GO:0005856 (cytoskeleton) was significantly different and associated with dendritic spines. Consequently, 10 noteworthy putative target genes of miR-142-5p were identified from the aforementioned pathway: SGCE, KITLG, USP9X, ELAVL4, SPIRE1, DMD, ACTN4, SLAIN1, RHOC, and PTPN4. Next, we examined the expression of these 10 genes. First, we stereotactically injected the miR-142-5p agonist (agomir) and NC control into the bilateral hippocampal region of normal rats and found that the mRNA levels of five target genes (ELAVL4, PTPN4, USP9X, ACTN4 and SLAIN1) were significantly reduced (Fig. 5B). Immediately after that, we examined the five differentially expressed genes in the hippocampal tissues of rats in both groups and found that the mRNA levels of ELAVL4, USP9X, and ACTN4 were significantly decreased in the hippocampal tissues of the CCI rats after 14 days of modeling than in sham rats, whereas the mRNA levels of PTPN4 and SLAIN1 were not significantly different (Fig. 5C, D).

In addition, we examined the protein levels of the above three important target genes in hippocampus, and found that the protein levels of ACTN4, ELAVL4 and USP9X were significantly lower in the rats’ hippocampusinjected bilaterally with the miR-142-5p agomir in the hippocampal stereotaxic space (24 h after the injection) than in NC control group (Fig. 5F); in contrast, compared to those in the sham group, the protein levels of ACTN4, ELAVL4 and USP9X were significantly lower in the hippocampus of CCI rats (Fig. 5G). Moreover, we examined the changes in the protein expression of target genes in hippocampus after the injection of AAV to interfere with miR-142-5p expression in Schwann cells and found that the protein levels of ACTN4, ELAVL4 and USP9X were significantly lower in the CCI + NC sponge group than in the Sham + NC sponge group. The miR-142-5p sponge significantly increased the protein expression of target genes in the hippocampus of CCI rats; in addition, AAV-miR-142-5p significantly reduced the protein expression of target genes in the hippocampus of CCI rats (Fig. 5H). These results suggest that ACTN4, ELAVL4 and USP9X were important target genes of miR-142-5p that are predominantly carried by SC-EVs.

### The overexpression of ACTN4, ACTN4 or ELAVL4 reverses mir-142-5p-mediated dendritic spine damage

**Fig. 6:**
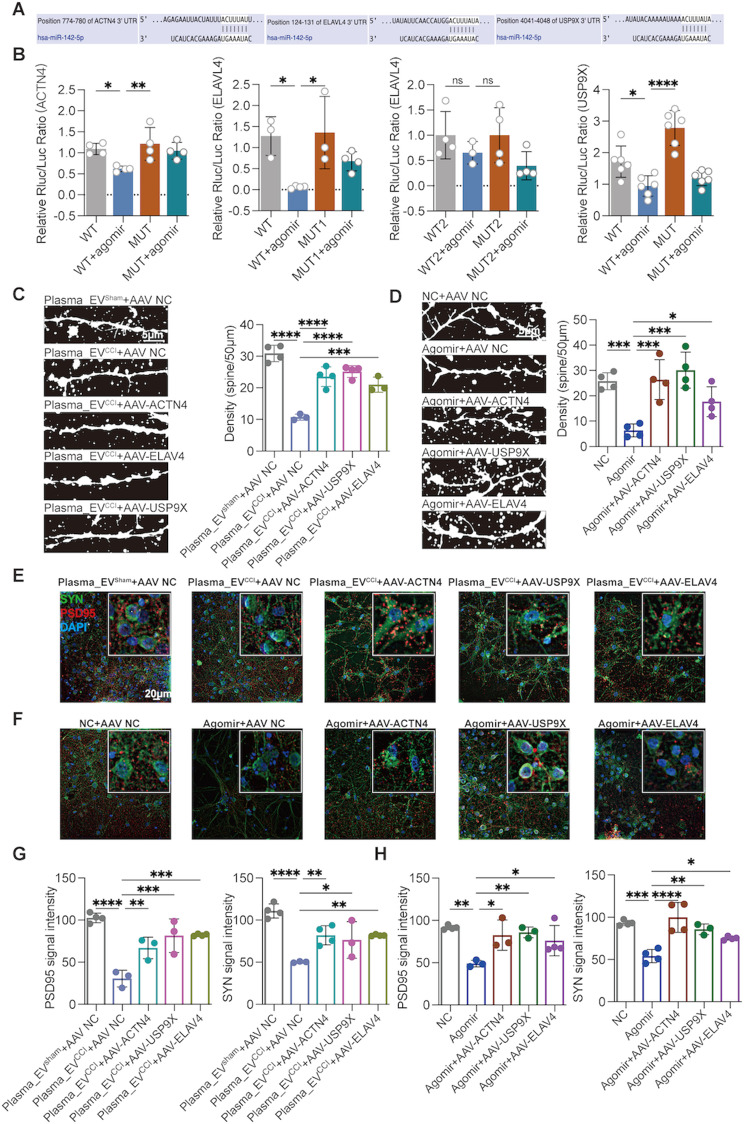
ACTN4, ELAVL4 and USP9X overexpression reversed miR-142-5p- and plasma_EV^CCI^-induced dendritic spine damage. (**A**) The potential binding sites of miR-142-5p in ACTN4, ELAVL4 and USP9X. (**B**) The direct effects of miR-142-5p on ACTN4, ELAVL4 and USP9X were identified by reporter gene analysis. miR-142-5p has three potential binding sites for ELAVL4; therefore, two mutant plasmids were designed separately for ELAVL4 (MUT1), one of which included two relatively similar binding sites (MUT2), as presented in Supplementary Fig. 8F. After transfecting HEK293T cells with miR-142-5p and reporters carrying the 3′UTRs (including mutated binding sites) of ACTN4, ELAVL4 and USP9X, dual-luciferase reporter activities were detected. Compared to the WT + agomir group, the miR-142-5p agomir group exhibited reversed Rluc/luc (Renilla luciferase activity) activity in response to the mutated reporter plasmids ACTN4, ELAVL4 (MUT1) and USP9X. ordinary one-way followed by Dunnett’s multiple comparisons test was used for ACTN4 and USP9X; ordinary one-way followed by Tukey’s multiple comparisons test was used for ELAVL4. (**C**–**H**) After transfecting primary hippocampal neurons with AAVs overexpressing ACTN4, ELAVL4 or USP9X, plasma EVs and the miR-142-5p agomir were coadministered, and DIL staining and immunofluorescence staining were performed. Overexpression of these genes significantly ameliorated the impairments in the dendritic spine density of neurons and the protein expression of PSD95 and SYN in the plasma_EV^CCI^ and miR-142-5p agomir groups compared with those in the plasma_EV^CCI^ and miR-142-5p agomir groups. *n* = 3–4 images from three wells of each group. ordinary one-way followed by Dunnett’s multiple comparisons test was used. The data are shown as the means ± SDs. *P < 0.05, **P < 0.01, ***P < 0.001, ****P < 0.0001

We predicted the binding partners of miR-142-5p with ACTN4, ELAVL4 and USP9X through a database. miR-142-5p has three binding sites for ELAVL4, two of which are in close proximity to each other; thus, two mutant plasmids, MUT1 and MUT2, were designed (containing two binding sites in close proximity). miR-142-5p has only one potential binding site for ACTN4 and USP9X (Fig. 6A and Supplementary Fig. 8F). To directly demonstrate that ACTN4, ELAVL4 and USP9X are direct downstream targets of miR-142-5p, we performed luciferase assays in HEK293T cells transfected with plasmids containing the predicted miR-142-5p binding site in the 3′ untranslated region (UTR). The use of the miR-142-5p agomir inhibited luciferase activity upstream of the 3′UTR of ACTN4, ELAVL4 and USP9X, and these inhibitory effects were rescued by the corresponding gene mutation (MUT1 of ELAVL4) (Fig. 6B). These results suggest that miR-142-5p can inhibit the expression of ACTN4, ELAVL4 and USP9X through binding.

Then, we further investigated the effects of ACTN4, ELAVL4 and USP9X on dendritic spines. We designed and constructed AAV overexpressing ACTN4, ELAVL4 and USP9X, respectively, transfected primary hippocampal neurons for 14 days, and then coadministered plasma EVs or the miR-142-5p agomir. As a result, we found that overexpression of ACTN4, ELAVL4 and USP9X, respectively, reversed the effects of plasma_EV^CCI^ or the miR-142-5p agomir on the dendritic spines of primary hippocampal neurons, as did the reversal of the reduction in spine density (Fig. 6C, D) and the associated reduction in the levels of the synaptic proteins PSD95 and SNY (Fig. 6E–H).

## Discussion

In the present study, we identified previously unknown mechanisms of sciatic nerve-brain interorgan communication in which Schwann cell-derived EVs and their cargo miRNAs can be transferred to the brain, particularly in the hippocampus, inducing dendritic spine remodeling and memory impairment in a CNP model. Inhibition of EV secretion and intervention with miR-142-5p ameliorated CNP-associated memory impairment and dendritic spine remodeling.

Exosomes (30–150 nm) and microvesicles are the most extensively studied extracellular vesicles [29]. Emerging data reveal that several forms of EVs, such as exosomes and microvesicles, can transfer functional proteins and RNA to surrounding or distant cells [34]. In addition, exosomes contain mRNAs and miRNAs that, when transported to destination cells, remain functional and change cellular behavior [34, 35]. EVs play a role in the etiology of various neurological disorders, including Alzheimer’s disease and Parkinson’s disease. However, studies have demonstrated that exosomes also regulate nociception and other sensory process pathways [36, 37]. Recent research has shown that exosomes isolated from the rat brain nucleus accumbens and medial prefrontal cortex contribute to allodynia and hyperalgesia following nerve damage [38]. Exosomes released by immune cells or stimulated SCs may be ingested by peripheral sensory neurons, triggering a chain reaction that results in neuronal sensitization. As a double-edged sword, SCs, critical peripheral nervous system components, are continually exposed to physiological and mechanical stressors during movement due to dynamic stretching and compression pressures [10, 11]. SC-EVs transmit cargo to promote peripheral nerve regeneration in vitro [11], but they also play a role in nociceptive hypersensitivity [39]. Here, we discovered for the first time that, compared to those in sham rats, SC-EVs in the hippocampus were significantly enriched in CNP rats by generating three classic models of CNP, namely CCI, SNI, and SNI (14 days after modeling), overexpressing specific SC promoters using AAVs containing CD63-GFP. Additionally, CD63-GFP signals were significantly enriched in the hippocampus (CA1-CA3) at 3, 7, 14, and 21 days after CCI modeling. As expected, treatment with the classical EV inhibitor GW4869 reversed the nociceptive hypersensitivity and memory impairment behaviors associated with CNP.

Moreover, although EVs contain proteins and lipids, they are highly enriched in noncoding RNAs. The miRNA content of EVs varies considerably according to the cell type of origin and does not just mirror the miRNA profile of donor cells [35]. Specific miRNAs are preferentially enriched in EVs. Protein and RNA exosome release has been postulated to constitute a fundamental method of communication in the nervous system, augmenting the established processes of anterograde and retrograde transmission across synapses [40, 41]. Disease states may alter exosome composition, and new research has revealed that SCI models of neuropathic pain affect the proteomic profile of sEVs in the mouse circulation [42]. An emerging finding showed that following peripheral nerve injury, sensory neurons transfer EV-encapsulated miR-23a to M1 macrophages, activating them and exacerbating neuropathic pain [37]. In this study, we found that CNP stress altered the composition of hippocampal EVs. By combining bioinformatic and in vivo approaches, we found that miR-142-5p is a common molecule that is significantly increased in the hippocampal tissues of CNP-associated memory impairment models. Furthermore, we demonstrated that the precursor and mature body mass of miR-142-5p are dramatically increased in the sciatic nerve. However, only the level of mature miR-142-5p was significantly increased in the hippocampal tissues of CNP model rats. Notably, an AAV carrying a specific Schwann cell promoter exacerbates or ameliorates CCI-associated memory impairment and hippocampal neuronal dendritic spine damage by upregulating or enclosing miR-142-5p expression in Schwann cell of sciatic nerve. In addition, intrasciatic nerve injections of the miR-142-5p antagomir also significantly attenuated CNP-associated memory impairment. miR-142-5p is implicated in neurodegenerative disorders such as Alzheimer’s disease, isoflurane-induced neurological impairment, and posttraumatic stress disorder, and inhibiting miR-142-5p improves memory impairment in these models [31, 32, 43]. An RNA sequencing profile of the sciatic nerve in the CCI model revealed that miR-142-5p levels were considerably greater in the CCI group than in the control group [44]. To our knowledge, our study is the first to report a critical role of Schwann cell-derived miR-142-5p in the development of memory impairment associated with CNP. These results provide an experimental basis for the therapeutic application of anti-miR-142-5p in CNP-associated memory disorders.

A new study revealed that ELAVL4 influences multiple biological pathways linked to Alzheimer’s disease, including those involved in synaptic function and the expression of genes downstream of APP and tau signaling [45]. Hu proteins participate in numerous aspects of posttranscriptional gene regulation by directly binding mRNAs, including mRNA polyadenylation, alternative splicing, trafficking, turnover, and translation [46]. ELAVL4 interacts with numerous unstable mRNAs at the molecular level, and as a consequence of this contact, the target transcript is stabilized [47]. ELAVL4 contains three RNA recognition structures, the first two necessary for binding to GAP-43 mRNA, one of ELAVL4’s best-studied targets [47]. In addition to GAP-43, other mRNAs, including BDNF, AChE, and tau, have been demonstrated to interact with ELAVL4 both in vitro and in vivo [48, 49].

Ankyrin-G contains multiple anchor protein repeat domains, and its isoforms are abundantly expressed in the brain and play important roles in a variety of neurobiological processes, including synaptogenesis, synaptic plasticity, action potential generation and transmission, and ion channel regulation, with the 190 kDa isoform being enriched in dendrites and postsynaptic densities and regulating dendritic spine structure. Usp9X can reduce the level of polyubiquitination of ankyrin-G and stabilize it to maintain dendritic spine development [20]. ACTN4 supports the transition from fine to mushroom spines and is required for metabotropic glutamate receptor-induced dynamic remodeling of dendritic protrusions [16]. We demonstrated for the first time that ACTN4, ELAVL4 and USP9X are direct target genes of miR-142-5p and that the mRNA and protein levels of ACTN4, ELAVL4 and USP9X in the hippocampus of CCI rats are decreased. Additionally, the expression of ACTN4, ELAVL4, and USP9X in the hippocampus of CCI rats was significantly downregulated by hippocampal stereotactic injection of a miR-142-5p agomir and specific AAV-mediated upregulation of miR-142-5p in Schwann cell. Conversely, the expression of these proteins was reversed by specific AAV-miR-142-5p sponge occlusion of miR-142-5p in Schwann cell. . In addition, in in vitro experiments, the antagomir ameliorated the inhibitory effect of plasma EVs on the expression of these proteins in primary hippocampal neurons from CCI rats. Additionally, dual-luciferase reporter gene analysis revealed that the 3’UTRs of ACTN4, ELAVL4 and USP9X could bind to miR-142-5p. Overexpression of these genes ameliorates dendritic spine damage and the inhibition of synaptic protein expression in primary rat hippocampal neurons via plasma_EV^CCI^ or miR-142-5p. Taken together, these results support the novel hypothesis that ACTN4, ELAVL4, and USP9X are important downstream molecules in Schwann cell-derived EVs that act as major carriers of miR-142-5p to mediate CNP memory impairment and dendritic spine damage in hippocampal neurons in the context of CNP.

Additionally, our study has several implications and limitations. We report a significant increase in both plasma extracellular vesicle concentration and particle size in CCI rats (modelled for 14 days) compared to the Sham group, which may suggest an effect of chronic neuropathic pain stress on the release of extracellular vesicles, as well as the potential for the number of plasma extracellular vesicles to be used as a biomarker of CNPP and cognitive impairment associated with CNPP. Existing studies have linked chronic inflammatory diseases to elevated EV concentrations and altered EV composition [50]. Some researchers have found that EV concentrations in plasma from a tibia fracture model (closely mimic complex regional pain syndrome), although comparable to controls, are significantly larger in particle size than in control mice [51]. Studies have found that circulating EV counts are significantly increased in patients with Myalgic encephalomyelitis/chronic fatigue syndrome (ME/CFS, debilitating disease with multiple symptoms, including pain, depression, and neurocognitive deterioration in function) and that circulating EV counts correlate significantly with serum C-reactive protein levels and have reported that circulating EV counts and EV-specific proteins can be used as novel biomarkers for the diagnosis of ME/CFS [52]. However, there are also studies that report no difference in the number of purified sEVs in the serum of mice four weeks after SNI, but there are differences in size [42]. In our study, the clinical value of extracellular vesicle contents was more emphasized and the phenomenon of changing extracellular vesicle concentrations may have been overlooked. Therefore, in future clinical studies, it is recommended to accurately quantify plasma extracellular vesicle levels and to assess the correlation with clinically relevant features of acute and chronic pain (e.g., inflammation, level of oxidative stress, or degree of pain and memory impairment) at different time points. Besides, whether and how pathological changes in plasma EV content, quantity, and size, which are altered in the context of CNP, contribute to CNP-associated memory impairment has not been addressed in recent studies. The pathological mechanisms underlying memory impairment associated with CNP are complex, and the molecular properties of EVs produced by other cell types in CNP model tissues, influenced by other pathological stresses affecting other cytopathologies in CNP, need to be clarified to fully understand the molecular processes involved in understanding memory impairment caused by chronic peripheral nerve injury.

Furthermore, we observed the same significantly increased GFP-CD63 signaling in both SNI and PSNL hippocampal regions (CA1 to CA3) compared to Sham, suggesting SC-EVs as a potential target for peripheral nerve injury. Future studies need to further explore the role of SC-EVs in peripheral nerve injury. Lastly, Neurological injury may lead to a maladaptive inflammatory response, with SCs and resident immune cells (e.g. mast cells and macrophages) being the first to respond, which ultimately also contributes to the development of persistent pain and the development of other complications such as memory impairment [53]. Several relevant studies have confirmed the link between peripheral inflammation and memory impairment [3]. In this study, we focused more on the role of SC-EVs and their contents in the transmission and communication with dendritic spines of CNS neurons. As EVs can also directly carry various cytokines, the future studies need to further investigate the mechanism of action of SC-EVs with neuroinflammation and cognitive impairment.

**Fig. 7:**
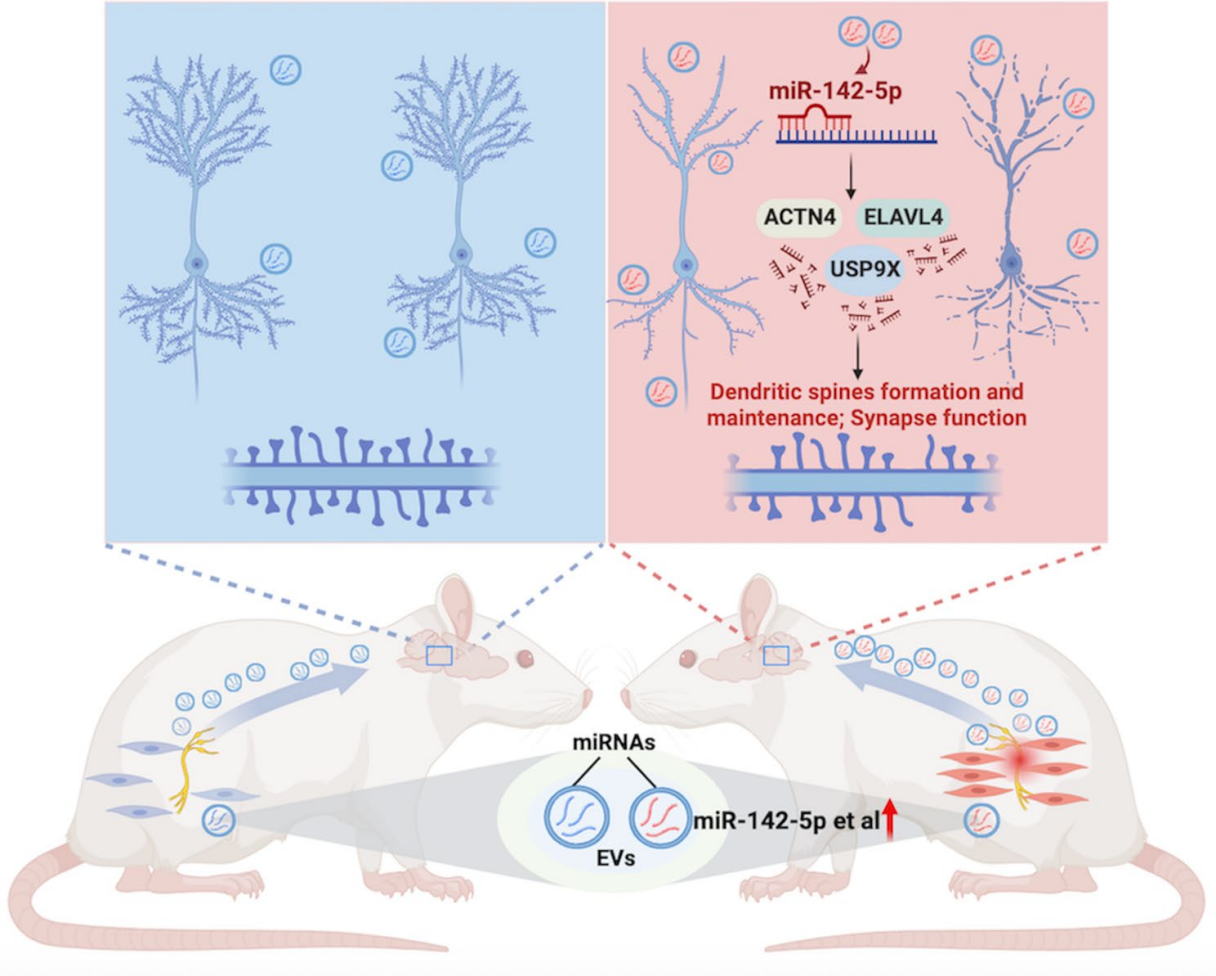
Schematic overview of the injured sciatic nerve-brain communication pathway that modulates dendritic spine remodeling in the hippocampus. Pathological Schwann cells-derived EVs and their cargo miR-142-5p mediate Schwann cells interorgan communication, inducing  dendritic spine damage and memory impairment associated with chronic neuropathic pain.

In summary, our study identified SC-EVs as novel mediators aggravating chronic peripheral nerve injury-associated dendritic spine modeling and memory impairment in the CNP model, as schematically illustrated in Fig. 7. These findings suggest that inhibiting aberrant SC-EV production, interfering with SC-derived miR-142-5p expression, and modulating hippocampal ACTN4, ELAVL4, and USP9X expression may be potentially practical therapeutic approaches for preventing and treating CNP-associated memory impairment.

### Electronic supplementary material

Below is the link to the electronic supplementary material.

Supplementary Material 1

Supplementary Material 2

Supplementary Material 3

Supplementary Material 4

Supplementary Material 5

Supplementary Material 6

Supplementary Material 7

Supplementary Material 8

Supplementary Material 9

Supplementary Material 10

## Data Availability

No datasets were generated or analysed during the current study.
