# Supplementary material for: Schwann cell-derived extracellular vesicles promote memory impairment associated with chronic neuropathic pain
Source: J Neuroinflammation. 2024 Apr 17;21:99. doi: 10.1186/s12974-024-03081-z (PMC11025217; doi:10.1186/s12974-024-03081-z)
Supplement: Supplementary file 1 — Supplementary Material 1 [file 12974_2024_3081_MOESM1_ESM.docx]

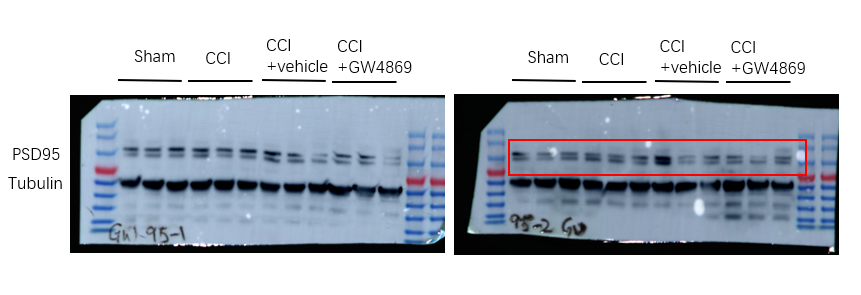


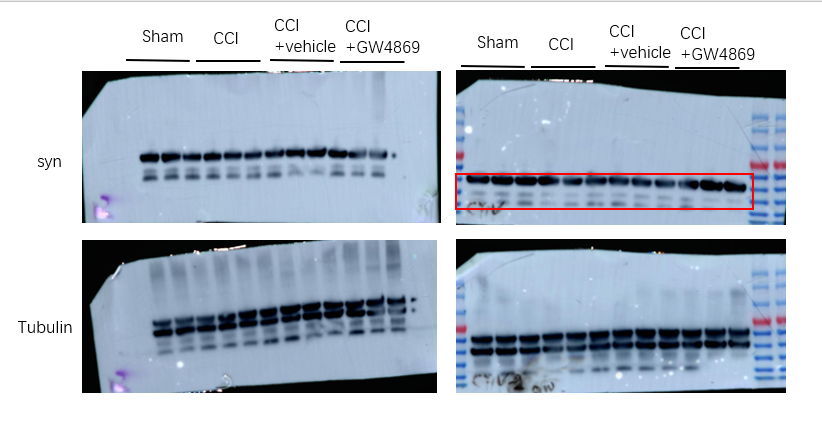


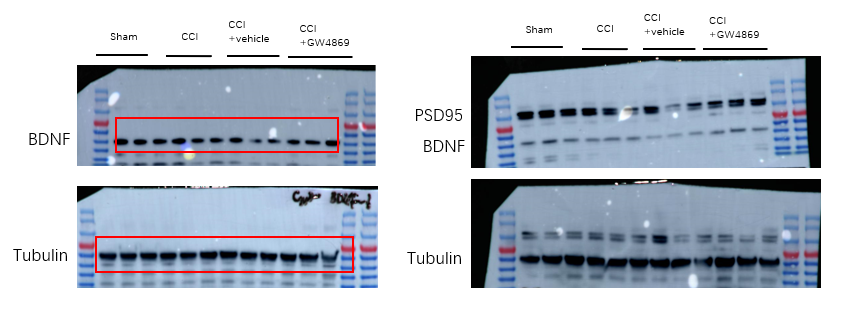


**Supplementary Figure 9**. Full and unprocessed western blot images corresponding to Figure 1, the square refers to the blots cited in the main article.


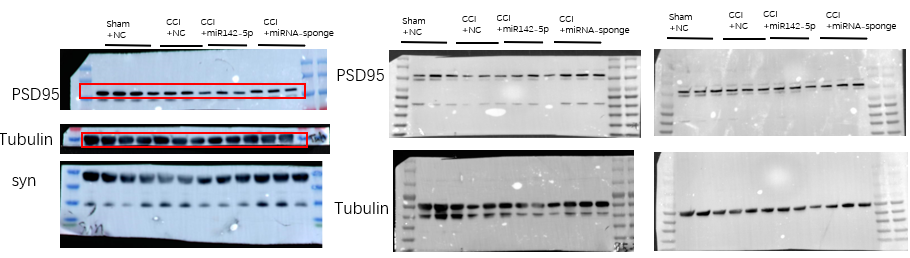


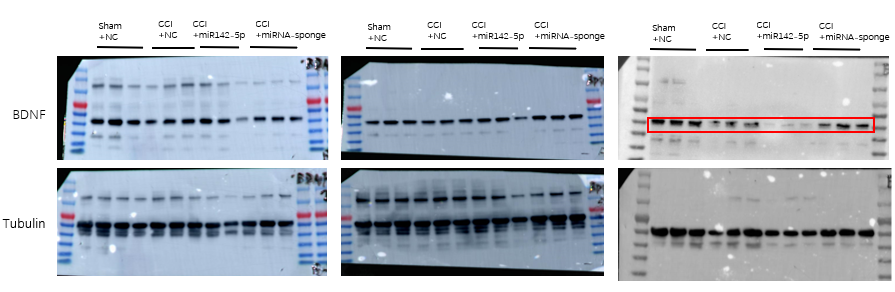


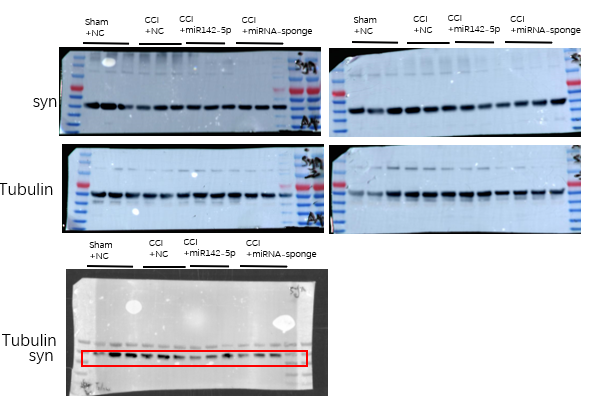


**Supplementary Figure 10**. Full and unprocessed western blot images corresponding to Figure 4G, the square refers to the blots cited in the main article.


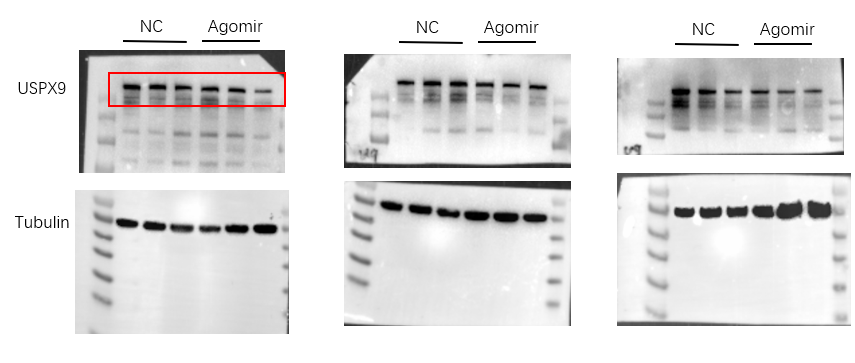


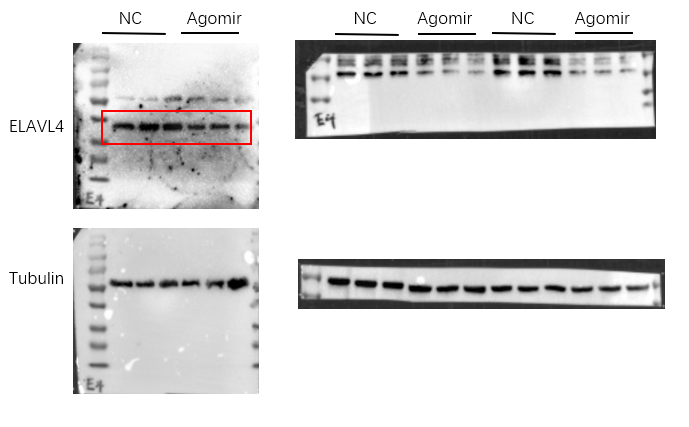


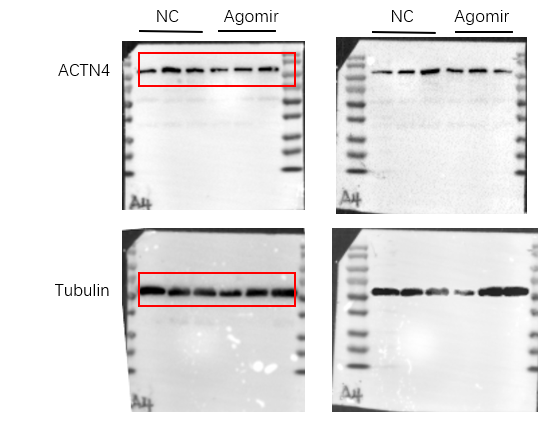


**Supplementary Figure 11**. Full and unprocessed western blot images corresponding to Figure 4F, the square refers to the blots cited in the main article.


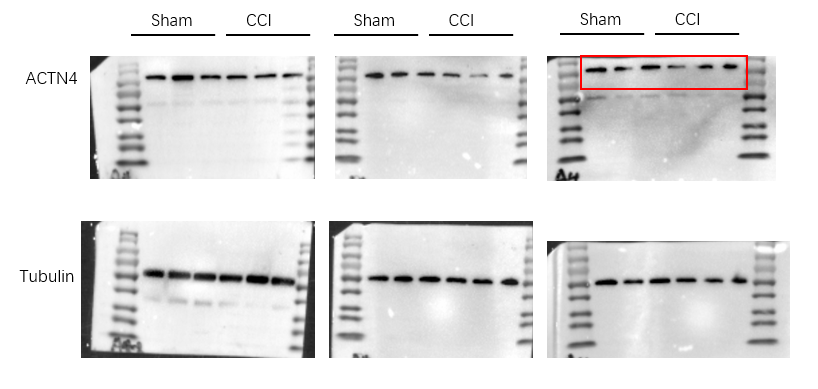


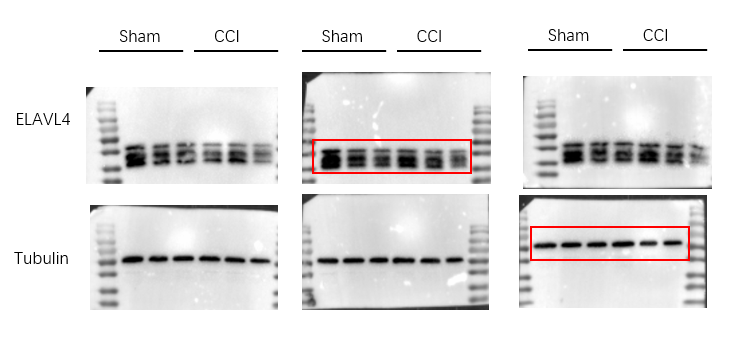


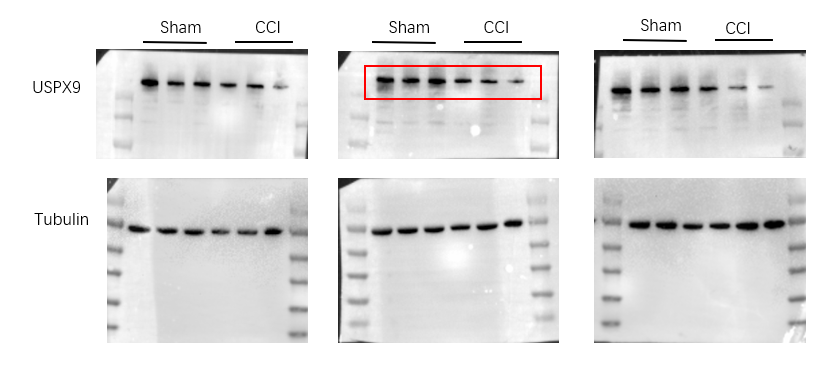


**Supplementary Figure 12**. Full and unprocessed western blot images corresponding to Figure 5G, the square refers to the blots cited in the main article.


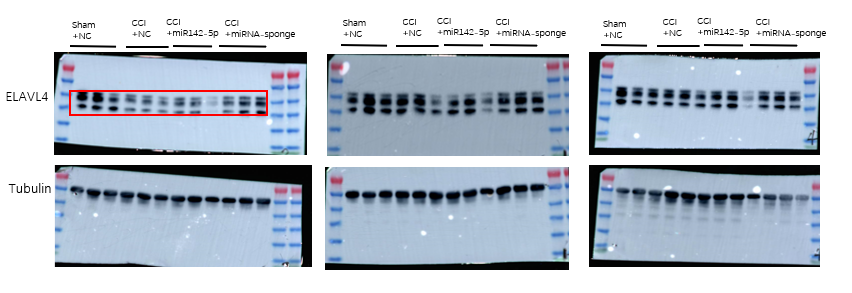


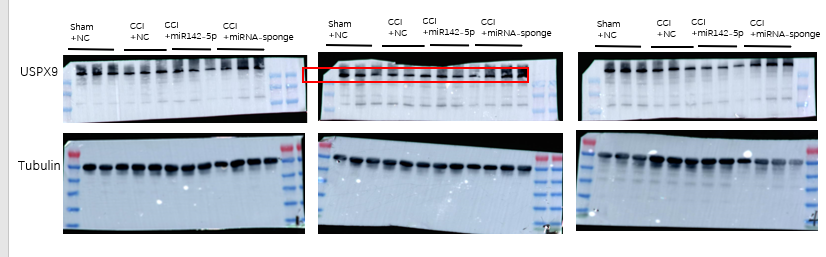


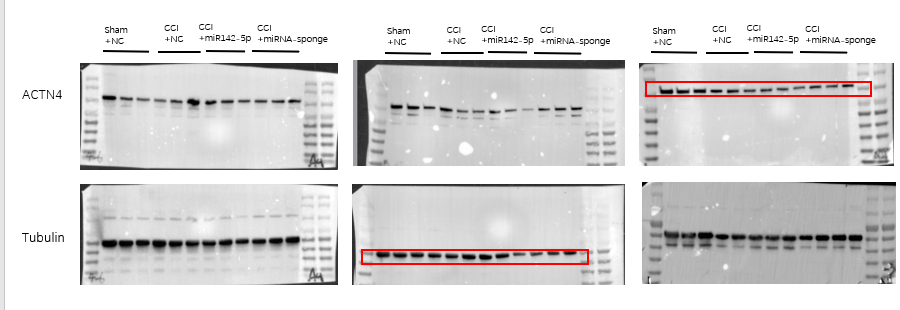


**Supplementary Figure 13**. Full and unprocessed western blot images corresponding to Figure 5H, the square refers to the blots cited in the main article.
