# Supplementary material for: Schwann cell-derived extracellular vesicles promote memory impairment associated with chronic neuropathic pain
Source: J Neuroinflammation. 2024 Apr 17;21:99. doi: 10.1186/s12974-024-03081-z (PMC11025217; doi:10.1186/s12974-024-03081-z)
Supplement: Supplementary file 2 — Supplementary Material 2 [file 12974_2024_3081_MOESM2_ESM.docx]

**Supplementary materials and methods information**

**Rat CNPP model establishment and treatment**

Rat models of CNPP induced by peripheral nerve injury were established as described previously [1, 2]. We independently established three traditional models of CNPP: chronic constriction injury (CCI) [1], spared nerve injury (SNI) [2], and partial sciatic nerve ligation (PSNL) [1]. Rats were anesthetized with 2%-3% isoflurane. The CCI model was established in which the left sciatic nerve and its branches were fully exposed and ligated four times above the proximal bifurcation of the sciatic nerve trunk with 4-0 chromium-containing intestinal thread to create a zone of chronic compression, subject to slight nerve sheath subluxation and left hind limb tremor. The SNI model was established as the left sciatic nerve was exposed, where it splits into the sural, tibial, and common peroneal nerves. The tibial and common peroneal nerves were first firmly ligated with 7.0-silk threads. Then, the nerves were severed immediately distal to the ligation, but the sural nerve was not affected in any way. For PSNL modeling, the sciatic nerve was exposed with scissors after the rear thighs were shaved bilaterally. The incision was then closed after the dorsal 1/3 to 1/2 of the nerve was gently ligated with 8-0 silk thread. The nerve was exposed (and closed) with no harm in the sham (no nerve injury) group. We successfully constructed a rat chronic neuropathic pain model using CCI, PSNL and SNI (Supplementary Fig. 1 and Supplementary Fig. 2). To inhibit exosome synthesis, intraperitoneal administration of the neutral sphingomyelinase-2 (nSMase2) inhibitor GW4869 (2 mg/kg, dissolved in saline containing 1% DMSO) was administered three times per week for three weeks in vivo [3].

**PWT, PWL, and acetone tests**

To evaluate mechanical allodynia, von Frey filaments were employed as previously reported[4]. Before the experiment, rats were placed in a clear glass frame (22*10*14 cm) for 30 minutes to acclimate. To assess paw withdrawal thresholds (PWTs), the up-and-down approach employs six carefully calibrated von Frey filaments. Von Frey filaments were applied to vertically stimulate the sole of the rat's hind paw (ranging from 0.008 to 300 g). Positive reactions comprised foot retraction, retreat, and paw licking. Thermodynamic hyperalgesia was measured using a previously described method [5]. Paw withdrawal latencies (PWLs) were measured using a thermal pain stimulator (Ugo Basile business). Three trials were performed on each rat at 5-minute intervals. Twenty seconds was chosen as the cutoff time for stimulation to avoid tissue damage. The cold test with acetone as the solvent was performed as detailed previously [6]. Fifty microliters of acetone were applied to the lateral plantar area of a hind paw. Amputation of the bisk foot was interpreted as a positive reaction. A paw withdrawal score and the duration of the withdrawal were both recorded.

**Y maze test**

To investigate spontaneous alternating behavior, the Y-maze test was performed as previously reported[7]. In brief, rats were placed in a Y-shaped contraption with three identical arms at a 120° angle labeled A, B, and C. Each animal was carefully placed in the center of the maze, which was then monitored and videotaped for 8 minutes with a video tracking device. Entering all three arms in the order ABC, BCA, and CAB was characterized as spontaneous alternation. The following is how the proportion of spontaneous alternations was calculated: The ratio of spontaneous alternations is calculated as follows: number of spontaneous alternations/(total number of arms - 2) * 100.

**Object recognition tests**

Object recognition tests, including the Object Recognition Test (ORM), Object Location Memory (OLM), and Temporal Order Memory (TOM), were carried out as previously reported. The experiments were conducted in a 60*60*50-centimeter box. Following habituation in the test chamber, rats were allowed to explore an empty arena for 5 minutes. After thirty minutes of investigation, the animals were given five minutes to explore the familiar arena with two similar items. After two hours, one of the two familiar items was transferred to a new position for the OLM. For ORM, the location of the objects remained the same, but one object was replaced with a new object. In addition, the TOM consisted of two training periods and a single test session. During each sampling phase, individuals were given 5 minutes to examine two identical items. If the rats' temporal memory were intact, they would have spent more time investigating sample 1's items. The discrimination index was calculated by dividing the total time spent exploring the two objects by the difference between exploring the new object/location and exploring the familiar object: ((time spent exploring the new object/location) - (time spent exploring the familiar object))/total time spent exploring.

**AAV vector construction, production and titration**

For EV tracing, we designed and built a pAAV2/8-Mpz promoter-Cd63-EGFP-3xFLAG-WPRE, referred to as AAV2/8-Mpz-CD63-GFP. Briefly, to track Schwann cell-derived EVs, GFP was fused to CD63 in vitro to fluorescently label EVs, which were then packaged into AAV2/8 containing the Schwann cell-specific promoter (Mpz). The viral vector was packaged and affinity purified by Obio Technology (Shanghai) Corp. Similarly, AAV9-hSyn promoter-MCS-EGFP-3FLAG-SV40 PolyA (AAV-hSyn-CD63-GFP) was conceived and produced for rat species utilizing the AAV9 serotype, overexpressing the CD63-GFP fusion gene, and containing a neuron-specific promoter to detect neuron-derived EVs (hSyn). Virus construction, packaging, and purification were performed by Shanghai Genechem Co. The fluorescent membrane protein was expressed on the membrane of EVs by coupling CD63 to a fluorescent protein (exosomes).

To overexpress miR-142-5p in Schwann cells, pAAV-Mpzpromoter-EGFP-3xFLAG-mir30(rno-miR-142-5p)-WPRE (AAV-miR-142-5p) was designed and built, and pAAV-CMV-EGFP-3xFLAG-WPRE served as the control. For the enclosure of miR-142-5p in Schwann cells, pAAV-Mpzpromoter-EGFP-3xFLAG-sponge(rno-miR-142-5p)-WPRE was designed and constructed, and pAAV-CMV-EGFP-3xFLAG-WPRE served as the control. To overexpress ACTN4, ELAVL4, and USP9X, AAVDJ-ACTN4, AAVDJ-ELAVL4 and AAVDJ-USP9X, respectively, were designed and constructed. Briefly, the target gene was cloned and inserted into a suitable viral vector, and then the recombinant expression plasmid was cotransfected with pHelper (carrying genes of adenoviral origin) and pAAV-RC (carrying genes for AAV replication and capsid) into AAV-293 cells. The AAV viral particles were collected, concentrated, filtered, purified, and finally packaged. Virus construction, packaging, and purification were performed by Obio Technology (Shanghai) Corp. and Shanghai Genechem Co.

**Separation of extracellular vesicles from tissue**

EVs were isolated from tissue using a protocol developed previously by Vella et al., with minor modifications[8]. The dissociation mixture was formulated utilizing the Miltenyi Human Tumor Dissociation Kit (Miltenyi Biotec, cat. no. 130-095-929). Before beginning, enzymes H, R, and A were resuspended as directed by the manufacturer. Before usage, a dissociation mixture consisting of 2.2 ml RPMI, 100 l enzyme H, 50 l enzyme R, and 12.5 l enzyme A was prepared. A tiny portion of tissue (200 mg) was weighed, thinly cut on dry ice, and incubated in the dissociation mixture for ten to fifteen minutes at 37°C. To eliminate residual tissues, the dissociated tissue was gently filtered twice through a 70 μm filter. After that, the suspension was spun at 300 × g for 10 minutes at 4°C, and the supernatant was transferred to a new tube and spun at 2000 × g for 10 minutes at 4°C. To further remove cell debris, the cell-free supernatant was centrifuged at 10,000 × g for 20 minutes at 4°C and then filtered slowly and gently through a 0.22 μm filter. The collected suspension was then ultracentrifuged at 150 000 × g for 2 hours at 4°C. The pellet was resuspended in 1 ml of phosphate-buffered saline (PBS) and purified using Exosupur® columns (Echobiotech, China). Amicon® Ultra spin filters (Merck, Germany) with a molecular weight cutoff of 100 kDa were used to concentrate the fractions to 200 μL.

Rat blood samples were taken from animal hearts. Blood (5-7 ml) was centrifuged at 1,000 × g for 20 minutes at 4°C to obtain plasma and at 10,000 × g for 1 h at 4°C to remove cells and cell debris. The clarified plasma was centrifuged at 100,000 × g for 60 min at 4°C. The isolated exosomes were resuspended in 500 μL of PBS and stored at −80°C for further experiments.

**Nanoparticle tracking analysis (NTA)**

The size and number of isolated particles were assessed using a ZetaView PMX 110 (Particle Metrix, Meerbusch, Germany) equipped with a 405 nm laser on vesicle suspensions with concentrations between 1x10^7^/ml and 1x10^9^/ml. Using NTA software, the motion of the particles in a movie of 60 seconds was captured at a frame rate of 30 frames per second (ZetaView 8.02.28).

**Transmission electron microscopy (TEM)**

The exosomes were contrasted with uranyl acetate solution for 1 minute after being washed with sterile distilled water. After that, the sample was dried for 2 minutes under incandescent lighting. A transmission electron microscope was used to study and photograph the copper mesh (H-7650, Hitachi Ltd., Tokyo, Japan).

**Scanning electron microscopy (SEM)**

A 10 μL solution of exosomes was deposited on a copper mesh and incubated at room temperature. EVs were placed onto silicon wafers and then dried in an oven. Prior to observation, a small coating of gold was sputter-coated onto the samples. Using a Hitachi S-3400 N electron microscope (Scanning Electron Microscope, FEI) with an acceleration potential of 15 kV, SEM images were acquired.

**Microbead-assisted flow cytometry**

Flow cytometry was conducted as previously described [9]. EVs from rat plasma were enriched on 4 μm aldehyde/sulfate latex beads (Invitrogen) by incubating 5 μg of EVs with 10 μL of beads for 15 min at room temperature with constant rotation. The suspension was then diluted with PBS to 500 μL and rotated at room temperature for 1 h. The reaction was halted with 100*10^-6^ m glycine and 0.5% BSA/PBS and rotated for 30 min at room temperature. The EV-bound beads were rinsed with 0.5% BSA in PBS and centrifuged for 3 min at 14,800 g, blocked with 5% BSA in PBS with rotation at room temperature for 1 h, washed with 0.5% BSA in PBS and centrifuged once more. The EV-bound beads were then incubated with rabbit anti-GFP antibody (Proteintech, Cat No. 66002-1-Ig) for 1 hour with rotation at 4 °C before being centrifuged for 3 minutes at 14 800 g. After discarding the supernatant, the beads were rinsed with 0.5% BSA/PBS and centrifuged for 3 minutes at 14 800 g. Next, the EV-bound beads were incubated for 30 minutes at 4 °C with secondary antibody (Alexa Fluor® 488 goat anti-rabbit IgG). Finally, the samples were washed three times with 0.5% BSA in PBS and resuspended in 200 μL of PBS. Flow cytometry analysis was conducted by a flow cytometer (Coulter CytoFLEX Flow Cytometer, Beckman).

**Immunofluorescence**

Rats were anesthetized with sodium pentobarbital (40 mg/kg, i.v.) and perfused through the ascending aorta with 0.9% saline (4°C) and 4% paraformaldehyde in PBS (4°C). After that, the whole brain, sciatic nerve, DRG, and spinal cord were removed and preserved in 4% paraformaldehyde for 24 hours before being dehydrated in 30% sucrose solution. Transverse brain slices (40 μm) were sliced using a frozen sectioning machine. Sections were initially blocked for 1 hour at room temperature in PBS containing 5% normal donkey serum and 0.1% triamcinolone acetonide. They were then incubated with primary antibodies overnight. After washing with PBS, the sections were incubated for 1 hour at room temperature with secondary antibody. Nuclei were stained with DAPI. Finally, the slices were examined blindly using an autoinverted fluorescence microscope (Olympus, Japan). All representative images and images used in the analysis were taken with the same confocal settings per experiment to allow for accurate comparisons of fluorescent intensity. ImageJ software determined the percentage of stained area for each chosen picture. For each rat tissue, three to five photos were selected at random. GFP (Proteintech, Cat No. 66002-1-Ig), CD63 (Abcam, Cat No. ab217345), MAP2 (Proteintech, Cat No. 17490-1-AP), S100β (Proteintech, Cat No. 66616-1-Ig), Iba-1 (Wako, Cat No. 011-27991), GFAP (Millipore, Cat No. MAB360), PSD95 (Cell Signaling Technology, Cat No. 36233), SYN (HUABIO, Cat No. ET1606-56), ACTN4 (Proteintech, Cat No. 19096-1-AP), ELAVL4 (Proteintech, Cat No. 67835-1-Ig), and USP9X (Proteintech, Cat No. 55054-1-AP) antibodies were used as primary antibodies.

**Western blot analysis**

Tissues were suspended in a lysis solution containing 50 mM Tris (pH 8.0), 150 mM NaCl, and protease inhibitor before being sonicated for 30 seconds at 10% amplitude (Ningbo Ultrasonic Cell Disruptor) to thoroughly solubilize total protein. Total proteins in the supernatant were extracted using a 16,000 g centrifuge at 4°C for 20 minutes. A BCA assay kit was used to assess the protein content of the supernatant (Beyotime, Nanjing, China). Protein samples were separated on polyacrylamide gels with a 15% polyacrylamide concentration and subsequently transferred to PVDF membranes (0.45 mm, Millipore, Bedford, MA, United States). Membranes were blocked with 5% skim milk (BD Biosciences) in Tris-buffered saline (TBST) containing 0.1% Tween for 1 hour at room temperature before being incubated overnight at 4°C with primary antibodies against mouse anti-PSD95 (Proteintech, Cat No. 20665-1-AP), rabbit anti-synaptophysin (ABclonal Technology, A6344), mouse anti-ELAVL4 (Proteintech, Cat No. 67835-1-Ig), mouse anti-ACTN4 (Proteintech, Cat No. 19096-1-AP), and mouse anti-USP9X (Proteintech, Cat No. 55054-1-AP). The membranes were incubated in TBST containing 5% skim milk for 1 hour with horseradish peroxidase-conjugated anti-rabbit IgG (goat, Abcam, ab6721). Immunoreactivity was identified using an enhanced chemiluminescence kit (Thermo Pierce, Waltham, MA, USA) and then analyzed using ImageJ. (Bio-Rad, United States). The expression levels of α-tubulin (Proteintech, Cat No. 11224-1-AP) were utilized to normalize the expression levels of the samples. The exosome supernatant was denatured in 5 × sodium dodecyl sulfonate (SDS) buffer and subjected to western blot analysis (10% SDS-polyacrylamide gel electrophoresis; 50 μg protein/lane) using rabbit polyclonal antibodies against CD63 (sc-5275, Santa Cruz, CA, USA), CD9 (60232-I-Ig, Proteintech, Rosemont, IL), HSP90 (60318-I-Ig, Proteintech, Rosemont, IL), Alix (sc-53540, Santa Cruz, CA, USA), TSG101 (sc-13611, Santa Cruz, CA, USA) and calnexin (10427-2-AP, Promega, Madison, WI).

**RT‒qPCR analysis**

Total RNA was isolated from samples using TRIzol reagent (Thermo Fisher Scientific, Waltham, MA, USA), as directed by the manufacturer. Relative expression (for mature miRNA) was determined using a miDETECT A Track miRNA qRT‒PCR Starter Kit (Ruibo, China). For genes and primary miRNAs, reverse transcription was performed. Real-time PCR assays were performed with HiScript® III RT SuperMix for qPCR (+gDNA wiper) (Vazyme Biotech Co., Ltd.) and Taq Pro Universal SYBR qPCR Master Mix (Vazyme Biotech Co., Ltd.) on a CFX96 Real-Time System (Bio-Rad Laboratories Inc., Hercules, CA, United States). 18 s was used as the gene and primary miRNA reference control, whereas U6 was used as the reference control for mature miRNA. Specific primer sets for miR-142-5p, miR-505-3p, miR-25-5p, miR-873-5p, and U6 were purchased from RiboBio. Other gene sequences of primers used are listed in Supplementary Table 1.

**Library preparation and sequencing**

According to the manufacturer's instructions, total RNA was isolated from exosomes using the miRNeasy Serum/Plasma Advanced Kit (Qiagen, cat. no. 217204). cDNA was subsequently synthesized from whole RNA using the PrimeScriptTM RT reagent Kit (Perfect Real Time) (TAKARA, RR037A). TaqMan® probe real-time qPCR was used to measure the target gene expression levels. Each PCR used μL of cDNA as its template. The RNA sample preparations for miRNA libraries employed 1 ng - 500 ng RNA per sample as input material. Using the QIAseq miRNA Library Kit (Qiagen, Frederick, MD) according to the manufacturer's instructions, sequencing libraries were produced, and index codes were added to ascribe sequences to each sample. To investigate the quantification of miRNA expression during cDNA synthesis and PCR amplification, reverse transcription (RT) primers with unique molecular indices (UMIs) were developed. Finally, the quality of the library was evaluated using the Agilent Bioanalyzer 2100 and qPCR. The index-coded samples were clustered on an acBot Cluster Generation System using TruSeq PE Cluster Kitv3-cBot-HS (Illumina, San Diego, California, United States) according to the manufacturer's instructions. After cluster creation, the library preparations were sequenced on an Illumina HiSeq platform to obtain paired-end reads.

**Quantification and differential expression analysis of miRNAs**

Using Bowtie tools soft, the clean reads corresponding to the Silva database, GtRNAdb database, Rfam database, and Repbase database sequence alignment were used to filter ribosomal RNA (rRNA), transfer RNA (tRNA), small nuclear RNA (snRNA), small nucleolar RNA (snoRNA), and other ncRNA and repetitions. The remaining readings were utilized to identify existing miRNAs and novel miRNAs predicted by comparing them to known miRNAs from miRbase and the Human Genome (GRCh38). EdgeR software was used to generate relative log expressions from the expression matrix of quantified UMI counts of miRNAs.

**Supplementary Table 1: qRT‒PCR primer sequences.**

| **Gene name** | **Forward** | **Reverse** |
| --- | --- | --- |
| SGCE | GGCTTAGGCAGGCCAAGAAT | TGCCATCGGCACAGCTTAAT |
| KITL | CTCAACTATGTCGCCGGGAT | CTTCGGTGCGTTTTCTTCCA |
| USP9X | GTCAAAGTCAGCGAAGTCCCC | ATTTGTTTTGCCTGTGGAGCA |
| ELAVL4 | GGGGCCTCCATCGCTTAC | TGTCTCTCACGAGTTTGCAGG |
| SPIRE1 | CCAGCTTGGCTGCATGAAGTT | TTATATGGACACCGACGCCATC |
| DMD | CCCGGAAAGCCAATGAGAGA | AACCATGCGGGAATCAGGAG |
| ACTN4 | TCGACAACAAGCACACCAACT | TGAAGTCATCTGAGTCCATGC |
| SLAIN1 | CCTGGCTTTCCCTTATAGCCC | TGGAAGCGTAGTCTTGCCTG |
| RHOC | CGGTGGAGCCCAAGTTTCA | CACCTGCTTGCCATCCACTT |
| PTPN4 | GACGCGGCTGTGATAACGAA | GATCCAGCCACCTGTTAACTCT |

**Supplementary Figure legends**

**Supplementary Figure 1. CCI model rats exhibit pain-related hyperalgesia and memory impairment.** (**A**) Flowchart for chronic neuropathic pain model construction and evaluation of nociceptive hypersensitivity and memory behaviors. (**B**) and (**C**) The paw withdrawal threshold (PWT), paw withdrawal latency (PWL), and cold pain score/duration were each determined using Von Frey filaments, thermal pain stimulators, and acetone tests, respectively. n = 12 rats, 2-way ANOVA followed by Šídák's multiple comparisons test. (**D**) Spontaneous alternating behaviors of rats at 7, 14 and 21 days after modeling were observed by the Y maze test. n = 12 rats, 2-way ANOVA followed by Šídák's multiple comparisons test. (**E**) and (**F**) Recognition memories in rats 21 days following modeling were assessed with the object recognition test (ORM), object location memory (OLM), and temporal order memory (TOM). n = 8-12 rats, unpaired Student’s t test. Data are shown as the means ± SDs. **p* < 0.05, ***p* < 0.01, ****p* < 0.001, *****p* < 0.0001.

**Supplementary Figure 2. Spared nerve injury (SNI) and partial sciatic nerve ligation (PSNL) model rats exhibit pain-related hyperalgesia and memory impairment.** (**A**) Flowchart for SNI and PSNL model construction and evaluation of nociceptive hypersensitivity and memory behaviors. (B) and (**C**) The paw withdrawal threshold (PWT), paw withdrawal latency (PWL), and cold pain score/duration were each determined using von Frey filaments, thermal pain stimulators, and acetone tests, respectively. n = 9-10 rats, 2-way ANOVA followed by Dunnett's multiple comparisons test. (**D**) Spontaneous alternating behaviors of rats at 7, 14 and 21 days after modeling were observed by the Y maze test, and recognition memories in rats 21 days following modeling were assessed with the object recognition test (ORM), object location memory (OLM), and temporal order memory (TOM). n = 9-10 rats, mixed-effect followed by Dunnett's multiple comparisons test for Y maze test, 2-way ANOVA followed by Dunnett's multiple comparisons test for ORM, OLM and TOM. Data are shown as the means ± SDs. **p* < 0.05, ***p* < 0.01, ****p* < 0.001, *****p* < 0.0001.

**Supplementary Figure 3. EV characterization, GW4869 improves pain in CCI rats, and successful AAV injection.** (**A**) to (**C**) Transmission electron microscopy, western blotting, and microbead-assisted flow cytometry revealed that the extracted EVs were nano distributed in EVs with particle sizes predominantly in the range of 50-300 nm and with typical vesicle structures and extracellular vesicle marker proteins. (**D**) When compared to the CCI or CCI+Vehicle group, GW4869 treatment significantly reduced the scores and duration of cold pain for CCI rats. n = 10 rats. 2-way ANOVA followed by Tukey's multiple comparisons test. (**E**) and (**F**) Immunofluorescence staining revealed that AAV was successfully injected into the sciatic nerve and DRG. Data are shown as the means ± SDs. **p* < 0.05, ***p* < 0.01, ****p* < 0.001, *****p* < 0.0001.

**Supplementary Figure 4. Immunofluorescence staining after AAV-CD63-GFP sciatic nerve injection.** (**A**) to (**D**) Little CD63-GFP signaling was found in the hippocampal DG, left sciatic nerve, left DRG, and spinal cord of rats in the three CNPP models (CCI, SNI, and PSNL) after AAV-CD63-GFP sciatic nerve injection.

**Supplementary Figure 5. Immunofluorescence staining after AAV-CD63-GFP sciatic nerve injection.** (**A**) Following AAV-CD63-GFP sciatic nerve injection, CD63-GFP signals were mostly absent in the cortical and prefrontal cortical regions of CCI animals. (**B**) to (**D**) Following AAV-CD63-GFP sciatic nerve injection, CD63-GFP signals were primarily colocalized with neurons (MAP2), with essentially minimal colocalization with microglia (Iba-1) or astrocytes (GFAP).

**Supplementary Figure 6. Immunofluorescence staining after AAV injection into the DRG.** We injected the DRG with AAV-hSyn-CD63-GFP containing a DRG-specific promoter and modeled it 14 days later. (**A**) to (**F**) Fourteen days after modeling, immunofluorescence labeling revealed virtually negligible CD63-GFP signals in the hippocampal CA1, CA2, CA3, and DG regions as well as cortical and prefrontal cortical regions.

**Supplementary Figure 7. Immunofluorescence staining after AAV-CD63-GFP sciatic nerve injection in various periods.** (**A**) to (**C**) At 3, 7 and 21 days after modeling, the hippocampal CA1/2/3 region of the CCI group showed richer GFP-CD63 colocalization signals than that of the sham group.

**Supplementary Figure 8.** (**A**) Blood‒brain barrier assay revealed increased blood‒brain barrier permeability in CCI rats compared to the sham group. (**B**) Scanning electron microscopy (SEM) indicated that the collected EVs were densely coated on the microbeads, indicating EV enrichment on the beads. (**C**) Flowchart for miRNA antagomir intervention and evaluation of nociceptive hypersensitivity and memory behaviors. (**D**) Spontaneous alternating behaviors of rats at 7, 14 and 21 days after modeling were observed by the Y maze test, and recognition memories in rats 21 days following modeling were assessed with the object recognition test (ORM), object location memory (OLM), and temporal order memory (TOM). n = 9-10 rats, 2-way ANOVA followed by Tukey's multiple comparisons test. (**E**) The paw withdrawal threshold (PWT), paw withdrawal latency (PWL), and cold pain score/duration were each determined using Von Frey filaments, thermal pain stimulators, and acetone tests, respectively. n = 9-10 rats, 2-way ANOVA followed by Tukey's multiple comparisons test. (**F**) Other binding sites of miR-142-5p to ELAVL4. Data are shown as the means ± SDs. **p* < 0.05, ***p* <0.01, ****p* <0.001, *****p* < 0.0001.

**References**

1. Bennett GJ, Xie YK: **A peripheral mononeuropathy in rat that produces disorders of pain sensation like those seen in man.** *Pain* 1988, **33:**87-107.

2. Fischer G, Pan B, Vilceanu D, Hogan QH, Yu H: **Sustained relief of neuropathic pain by AAV-targeted expression of CBD3 peptide in rat dorsal root ganglion.** *Gene Ther* 2014, **21:**44-51.

3. Gan L, Xie D, Liu J, Bond Lau W, Christopher TA, Lopez B, Zhang L, Gao E, Koch W, Ma XL, Wang Y: **Small Extracellular Microvesicles Mediated Pathological Communications Between Dysfunctional Adipocytes and Cardiomyocytes as a Novel Mechanism Exacerbating Ischemia/Reperfusion Injury in Diabetic Mice.** *Circulation* 2020, **141:**968-983.

4. Chaplan SR, Bach FW, Pogrel JW, Chung JM, Yaksh TL: **Quantitative assessment of tactile allodynia in the rat paw.** *J Neurosci Methods* 1994, **53:**55-63.

5. Hargreaves K, Dubner R, Brown F, Flores C, Joris J: **A new and sensitive method for measuring thermal nociception in cutaneous hyperalgesia.** *Pain* 1988, **32:**77-88.

6. Tang Y, Liu C, Zhu T, Chen H, Sun Y, Zhang X, Zhao Q, Wu J, Fei X, Ye S, Chen C: **Transcriptome Profiles of IncRNA and mRNA Highlight the Role of Ferroptosis in Chronic Neuropathic Pain With Memory Impairment.** *Front Cell Dev Biol* 2022, **10:**843297.

7. Zhang GF, Zhou ZQ, Guo J, Gu HW, Su MZ, Yu BC, Zhou F, Han BY, Jia M, Ji MH, et al: **Histone deacetylase 3 in hippocampus contributes to memory impairment after chronic constriction injury of sciatic nerve in mice.** *Pain* 2021, **162:**382-395.

8. Vella LJ, Scicluna BJ, Cheng L, Bawden EG, Masters CL, Ang CS, Willamson N, McLean C, Barnham KJ, Hill AF: **A rigorous method to enrich for exosomes from brain tissue.** *J Extracell Vesicles* 2017, **6:**1348885.

9. Wenzhe L, Bin S, Changliang L, Huayi W, Wangshu Z, Weiyao K, Xiaoran L, Guobin X, Chen W, Huiping L: **Noninvasive Diagnosis and Molecular Phenotyping of Breast Cancer through Microbead-Assisted Flow Cytometry Detection of Tumor-Derived Extracellular Vesicles.** *Small Methods* 2018**:**1800122.
