# Supplementary figures and images for: Schwann cell-derived extracellular vesicles promote memory impairment associated with chronic neuropathic pain
Source: J Neuroinflammation. 2024 Apr 17;21:99. doi: 10.1186/s12974-024-03081-z (PMC11025217; doi:10.1186/s12974-024-03081-z)

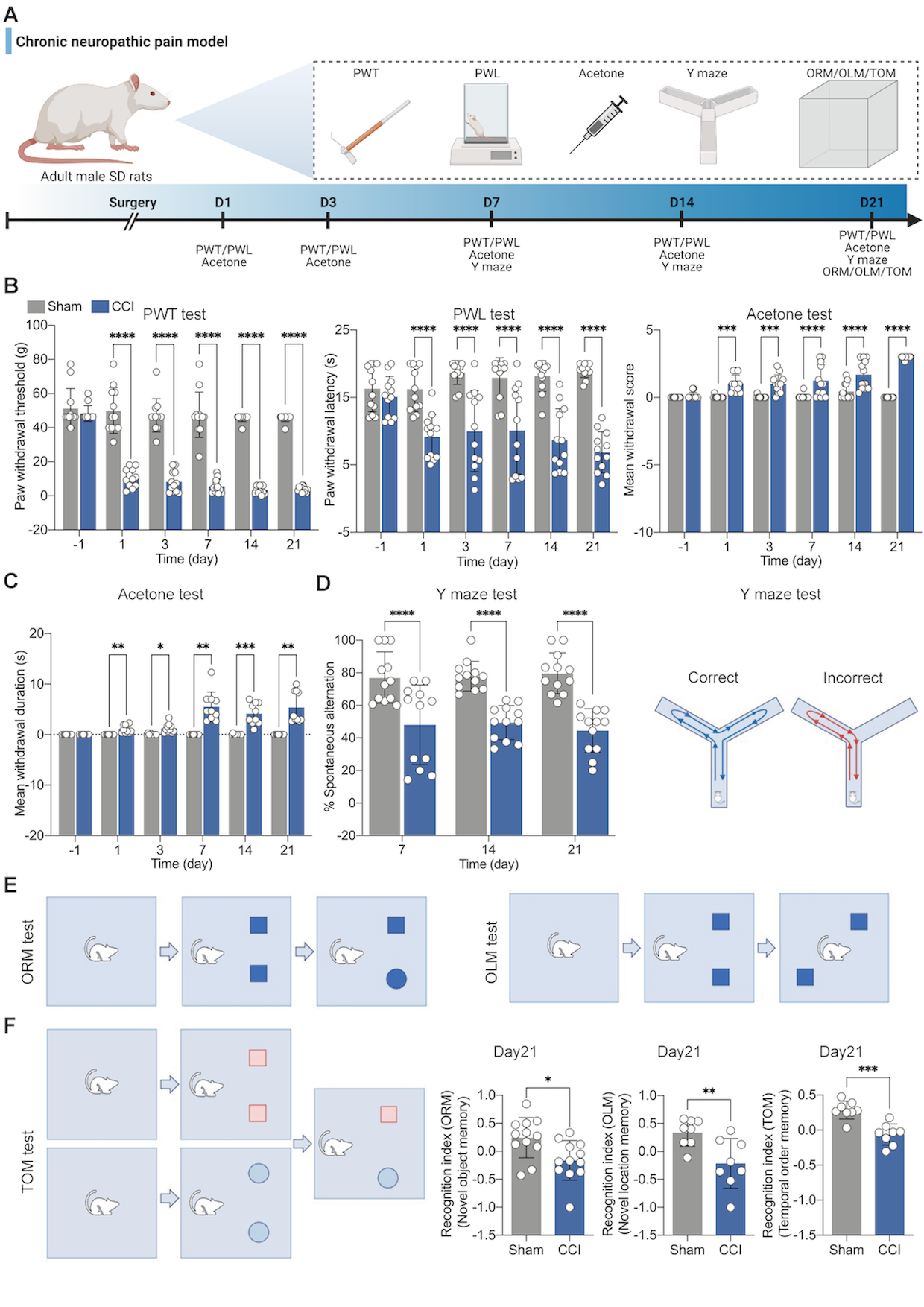

Supplement: Supplementary file 3 — Supplementary Material 3 [file 12974_2024_3081_MOESM3_ESM.tiff]

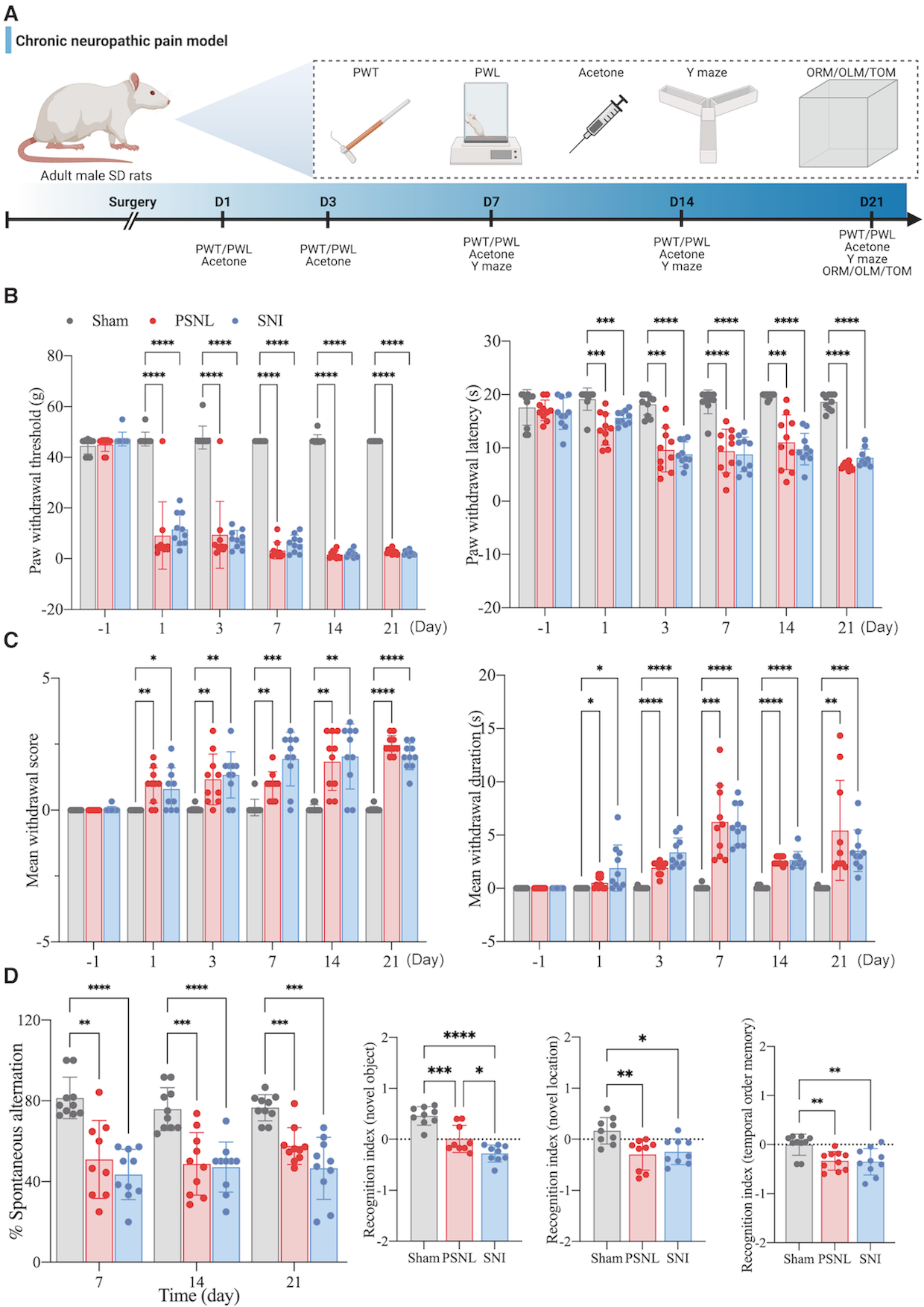

Supplement: Supplementary file 4 — Supplementary Material 4 [file 12974_2024_3081_MOESM4_ESM.tiff]

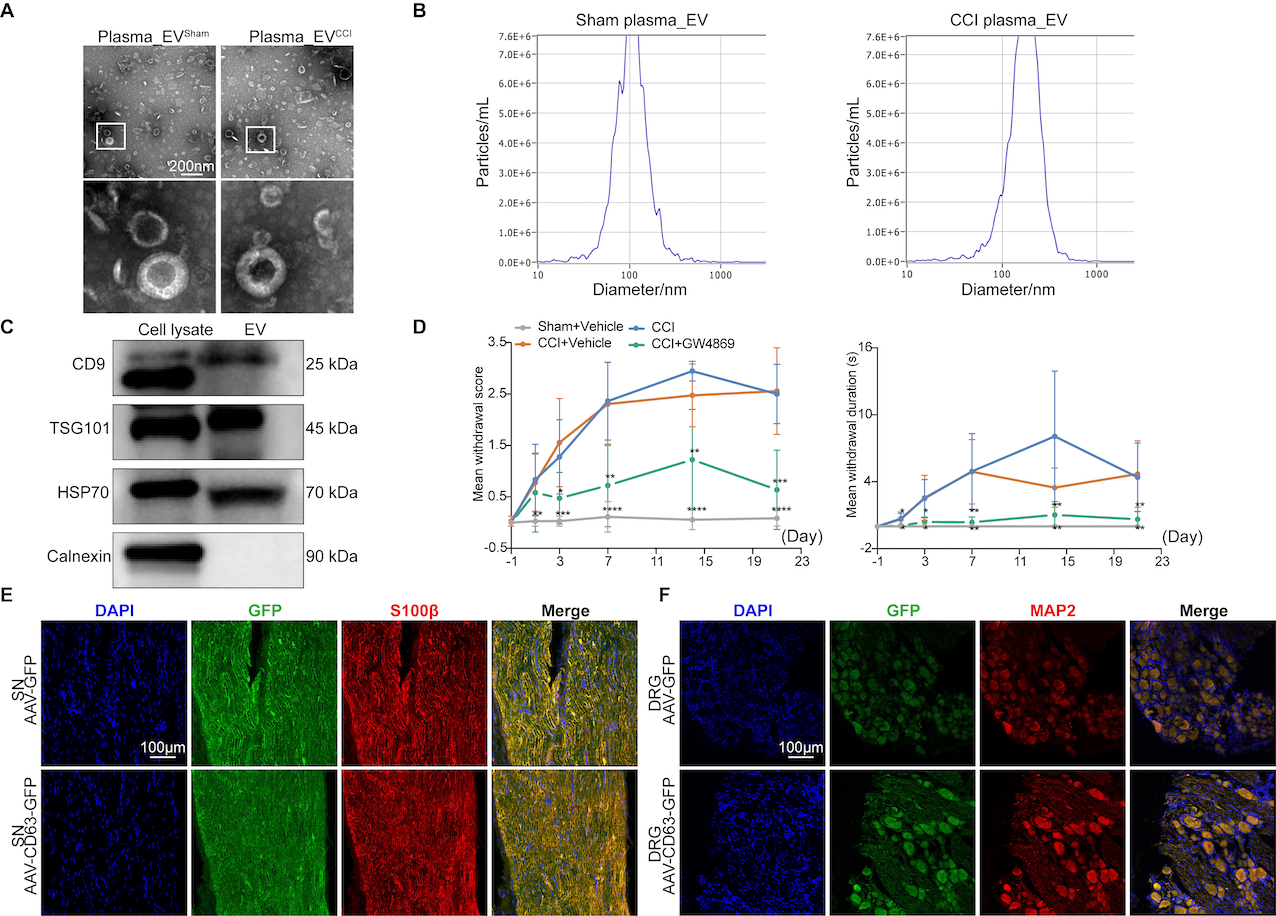

Supplement: Supplementary file 5 — Supplementary Material 5 [file 12974_2024_3081_MOESM5_ESM.tif]

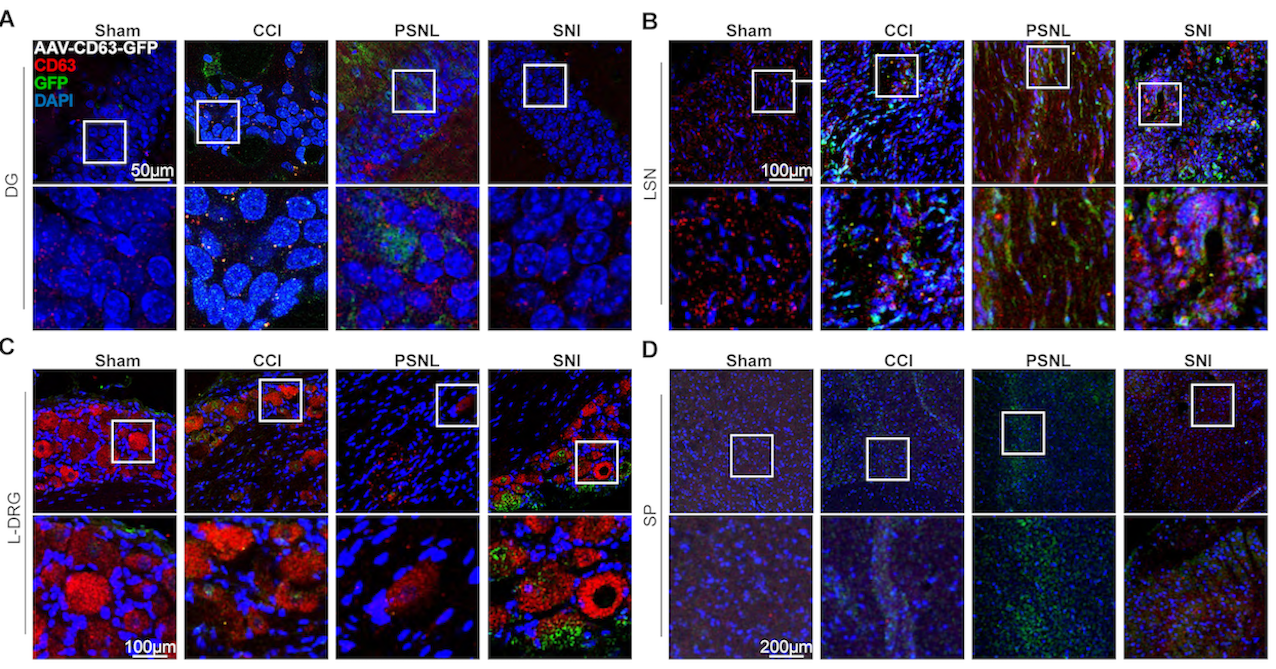

Supplement: Supplementary file 6 — Supplementary Material 6 [file 12974_2024_3081_MOESM6_ESM.tiff]

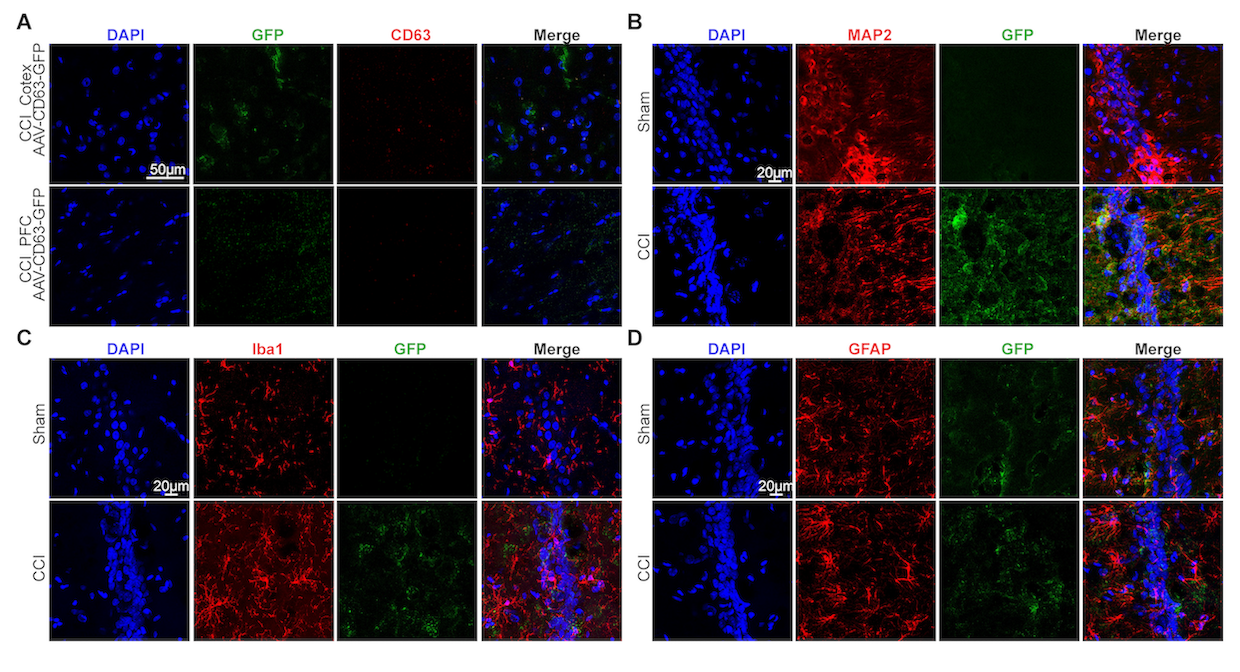

Supplement: Supplementary file 7 — Supplementary Material 7 [file 12974_2024_3081_MOESM7_ESM.tiff]

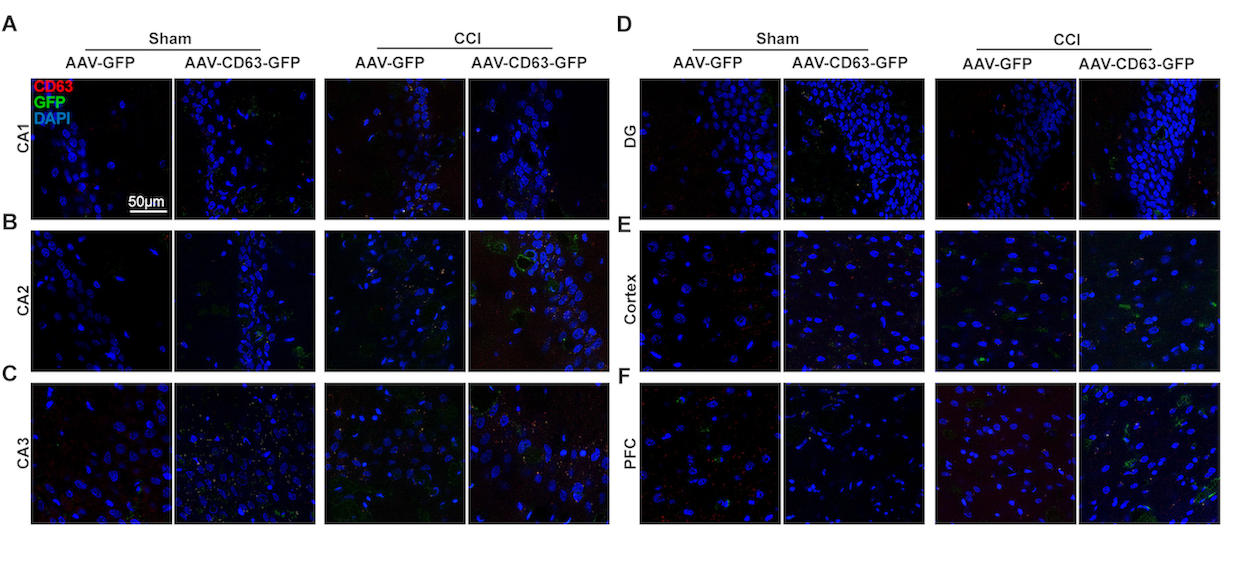

Supplement: Supplementary file 8 — Supplementary Material 8 [file 12974_2024_3081_MOESM8_ESM.tiff]

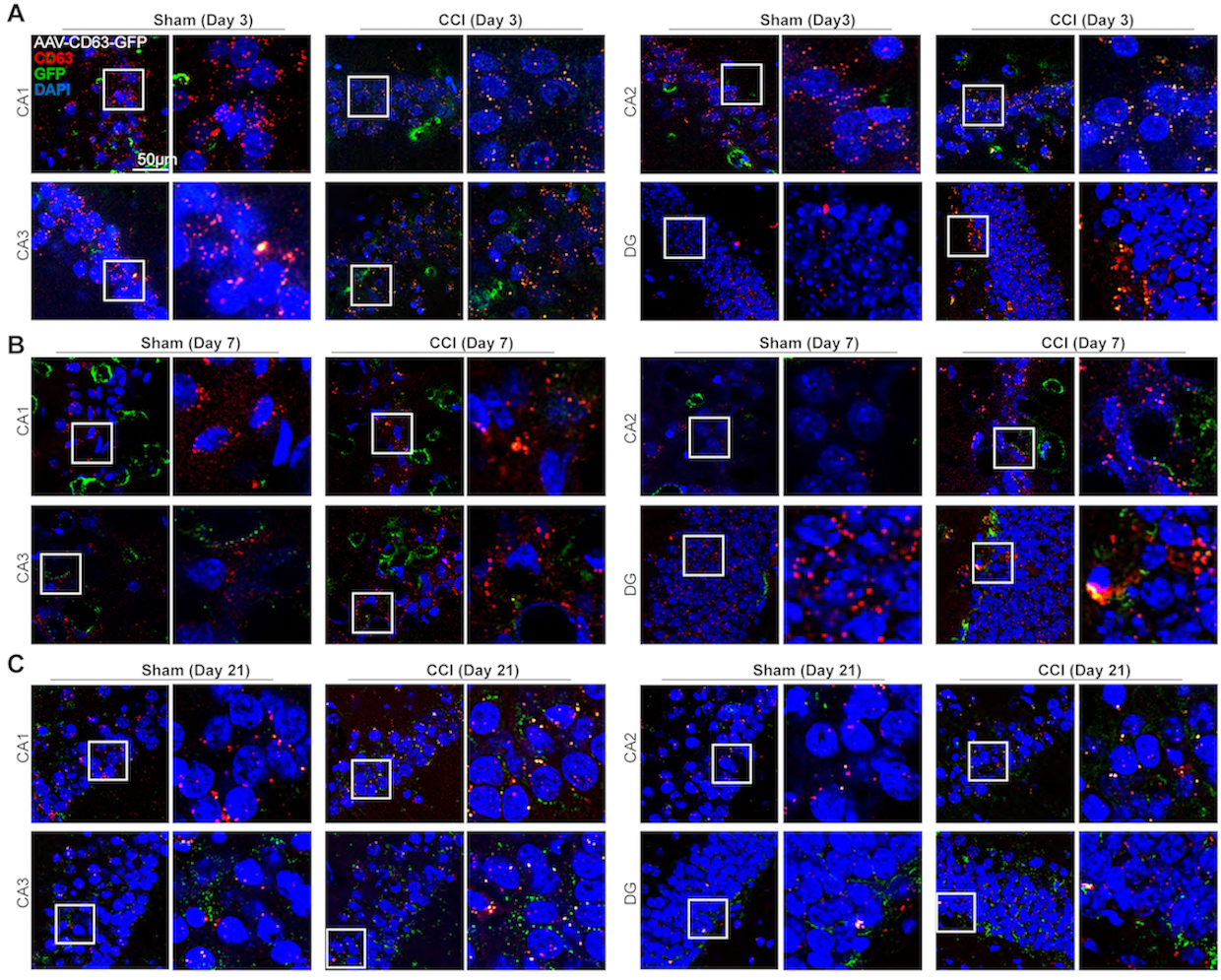

Supplement: Supplementary file 9 — Supplementary Material 9 [file 12974_2024_3081_MOESM9_ESM.tiff]

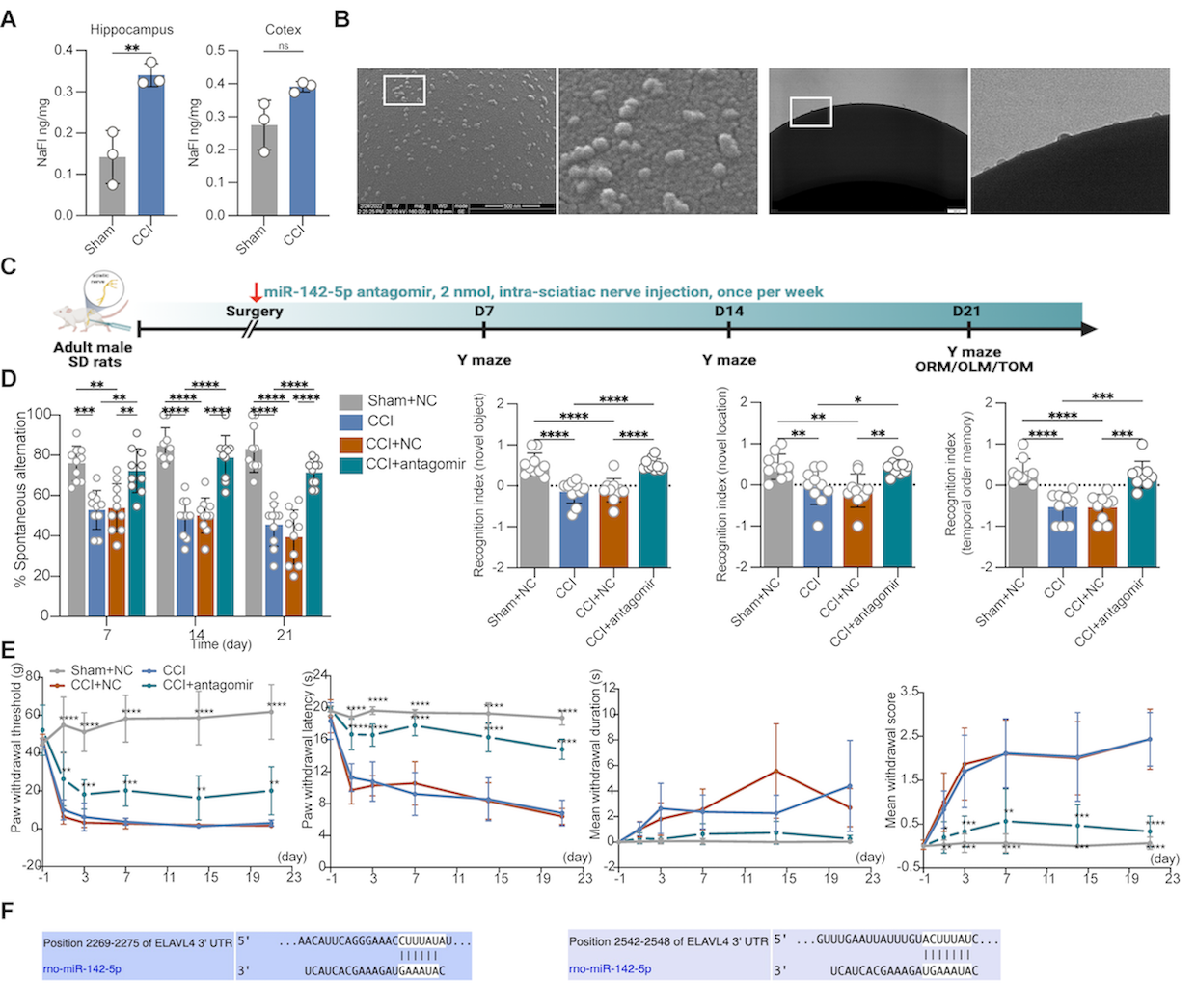

Supplement: Supplementary file 10 — Supplementary Material 10 [file 12974_2024_3081_MOESM10_ESM.tiff]
